# Evidence for [Coronal] Underspecification in Typical and Atypical Phonological Development

**DOI:** 10.3389/fnhum.2020.580697

**Published:** 2020-12-22

**Authors:** Alycia E. Cummings, Diane A. Ogiela, Ying C. Wu

**Affiliations:** ^1^Department of Communication Sciences and Disorders, Idaho State University, Meridian, ID, United States; ^2^Swartz Center for Computational Neuroscience, University of California, San Diego, San Diego, CA, United States

**Keywords:** ERP, underspecification, MMN, phonology, children, phonological disorder

## Abstract

The Featurally Underspecified Lexicon (FUL) theory predicts that [coronal] is the language universal default place of articulation for phonemes. This assumption has been consistently supported with adult behavioral and event-related potential (ERP) data; however, this underspecification claim has not been tested in developmental populations. The purpose of this study was to determine whether children demonstrate [coronal] underspecification patterns similar to those of adults. Two English consonants differing in place of articulation, [labial] /b/ and [coronal] /d/, were presented to 24 children (ages 4–6 years) characterized by either a typically developing phonological system (TD) or a phonological disorder (PD). Two syllables, /bɑ/ and /dɑ/, were presented in an ERP oddball paradigm where both syllables served as the standard and deviant stimulus in opposite stimulus sets. Underspecification was examined with three analyses: traditional mean amplitude measurements, cluster-based permutation tests, and single-trial general linear model (GLM) analyses of single-subject data. Contrary to previous adult findings, children with PD demonstrated a large positive mismatch response (PMR) to /bɑ/ while the children with TD exhibited a negative mismatch response (MMN); significant group differences were not observed in the /dɑ/ responses. Moreover, the /bɑ/ deviant ERP response was significantly larger in the TD children than in the children with PD. At the single-subject level, more children demonstrated mismatch responses to /dɑ/ than to /bɑ/, though some children had a /bɑ/ mismatch response and no /dɑ/ mismatch response. While both groups of children demonstrated similar responses to the underspecified /dɑ/, their neural responses to the more specified /bɑ/ varied. These findings are interpreted within a proposed developmental model of phonological underspecification, wherein children with PD are functioning at a developmentally less mature stage of phonological acquisition than their same-aged TD peers. Thus, phonological underspecification is a phenomenon that likely develops over time with experience and exposure to language.

## Introduction

Accurate speech perception is a complex process (Aslin and Smith, 1988). For example, auditory sensory information must first be detected, and then transformed into a neural representation of the event, with meaning eventually attributed to the auditory input. More specifically, phonological representations are formed by decoding of the speech signal, which requires, in part, the extraction and sequencing of phonetic features from the auditory signal (Scott and Wise, 2004). Being able to accurately perceive speech sounds allows for the accurate formation of phonemic categories, and more importantly, the accurate identification of words. Thus, it is important to form detailed phonological representations so that accurate speech production can occur.

Children appear to be born with the capability of differentiating [nearly] all the sounds of human speech (Eimas et al., 1971; Eimas, 1975). However, within the first year of life, infants' phonetic sensitivity decreases as they attend to the statistical distributions of sounds in the input (Kuhl, 2000, 2010). For example, infants can discriminate native vowel phonemes by 6 months of age and native consonants by 10 months of age (Werker and Tees, 1984; Kuhl et al., 1992; Werker and Hensch, 2015). Thus, while young children have the ability perceive subtle differences in sounds, it is only with time and language experience that they assign phonological meaning to the sounds. This suggests that over time, children learn which features are necessary for phonemic categorization in their native language(s) (Cheour et al., 1998; Kuhl et al., 2008).

Together, these findings suggest that auditory speech perception changes during phonological development (Nittrouer and Miller, 1997). Initially, sounds can be perceived and differentiated, but are not necessarily assigned to a phonological representation. Sound is given phonological meaning only after features are identified and categorized into distinct phonological representations. These phonological representations subsequently change how sound is perceived, as the auditory system goes from identifying all sound distinctions to making distinctions that are relevant to a given language. It is possible that children initially perceive differences between a wide variety of phonetic features in speech sounds, but as they develop the phonological representations for their language, they become less sensitive to those features that are irrelevant or redundant and may assign default status to those that are most frequent. Thus, as children develop their phonological representations, they may not yet be refined to adult-like levels. Children could initially store redundant features in their phonological representations, as they have not yet learned those features are unnecessary for phonemic categorization. As a result, some children's phonological representations might contain more features than those of adults.

Being able to accurately perceive speech sounds allows for the accurate formation of phonemic categories and the accurate identification of words. That being said, speech perception and production must also be efficient (Chomsky and Halle, 1968; Eulitz and Lahiri, 2004). Having to access and process extremely detailed representations of each phoneme would likely be inefficient for rapid speech processing. Moreover, it is questionable whether all features need to be stored in a phonological representation. One proposed solution to this problem is phonological underspecification, during which only the contrastive or not otherwise predictable phonological information (i.e., distinctive features) is stored for each phoneme (Kiparsky, 1985; Archangeli, 1988; Mohanan, 1991; Steriade, 1995; Hestvik and Durvasula, 2016).

While underspecification is proposed to be a language-universal phenomenon, the course of its development is presently unknown. As a language universal, is it present from birth? Or, is it something that develops over time, similar to the acquisition of phonemic categories? One aspect of phonological development could involve the establishment of specified and underspecified phonemes. Bernhardt (1992) proposed that children first develop and define the phonological role of underspecified features and then gradually define the phonological role of more specified features. For example, the [coronal] consonantal place of articulation feature (i.e., produced with tongue tip or blade, such as /t/ or /d/) is typically assumed to be less specified than other places of articulation (e.g., [labial] (i.e., produced with lips, such as /p/ or /b/) or [dorsal] (i.e., produced with dorsum of tongue, such as /k/ or /ɡ/) (e.g., Eulitz and Lahiri, 2004; Cornell et al., 2011, 2013; Cummings et al., 2017). Given that [coronal] is the proposed default place of articulation, children should acquire this underspecified place of articulation early in development. If children slowly acquire specified features, more marked place of articulation features such as [labial] would become established at a later age. As a result, children are predicted to first acquire the least specified phonemes, and more specified phonemes are added over time as features are defined and categorized. Thus, American English-speaking children are expected to produce phonemes with 90% accuracy by the following ages: 2;11 (years; months)—/p b d m n h w/; 3;11—/t k ɡη f j/ 4;11—/v s z ʃ ʧ ʤ l/; 5;11—/ʒ 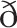 ɹ/; and 6;11—/θ/ (Crowe and McLeod, 2020).

While behavioral studies have successfully identified children's categorical speech perception abilities, behavioral tasks offer little if any insight into their underlying phonological representations. However, neuroimaging tools have proven useful in examining phonological underspecification. Neural markers of phonological underspecification have primarily been examined using the framework established by the Featurally Underspecified Lexicon (FUL) model (Lahiri and Marslen-Wilson, 1991; Lahiri and Reetz, 2002, 2010).

FUL predicts asymmetries in speech processing when an underspecified phoneme is contrasted with a more fully specified phoneme. A sound can directly *match* when the features extracted from the acoustic signal are the same as those in the phonological representation. A sound would be a *mismatch* when the features extracted from the acoustic signal are distinct from those in the phonological representation. A sound is a *no-mismatch* when the features extracted from the acoustic signal are consistent with the phonological representation, but because the phonological representation is not specified for a certain feature present in the speech signal, the input and the representation cannot exactly match (Schluter et al., 2016).

FUL's predictions have been tested using electrophysiological methodologies, such as event-related potentials (ERPs) that often measure mismatch negativity (MMN) responses (Näätänen et al., 2007). The MMN is an attention-independent neurophysiological response elicited by an acoustically different (deviant) stimulus when presented in a series of homogenous (standard) stimuli. Thus, the MMN is an automatic auditory change detection response in the brain and is thought to reflect stimulus discrimination and sensory memory (Sams et al., 1985); it is elicited by any discriminable acoustic contrast. The MMN is sensitive to language-specific speech sound representations (Näätänen et al., 1997; Kraus et al., 1998; Winkler et al., 1999; Phillips et al., 2000; Näätänen, 2001). Indeed, there is evidence to suggest that the MMN response is deviant in children with known language disabilities in response to tones (Korpilahti and Lang, 1994; Rinker et al., 2007; Ahmmed et al., 2008) and to speech syllables (Kraus et al., 1996; Uwer et al., 2002; Shafer et al., 2005; Volkmer and Schulte-Körne, 2018). Importantly, the MMN has been shown to index a person's ability to *behaviorally* discriminate between standard and deviant stimuli (Sams et al., 1985; Kraus et al., 1996). Children with better phoneme processing abilities have demonstrated larger MMN responses than children scoring lower on a phoneme processing test (Linnavalli et al., 2017).

In terms of underspecification, the MMN varies depending on whether the specified or underspecified phoneme is the standard (and deviant), as the standard stimulus sets up the feature expectations that the deviant stimulus will match, mismatch, or no-mismatch. In the match condition, the same feature is present in the deviant and standard stimuli, resulting in no MMN response. In the no-mismatch condition, the underspecified phoneme is the standard stimulus, which does not set up a feature expectation for the more specified deviant stimulus. As a result, little or no MMN response is expected in the no-mismatch condition. The true mismatch condition occurs when the more specified phoneme is the standard stimulus and sets up a specific feature expectation for the less specified deviant stimulus. The feature extracted from the deviant stimulus signal directly conflicts with that of the standard, resulting in a large MMN response.

FUL predicts that [coronal] phonemes have the default place of articulation because they contain less distinctive feature information in their phonological representations than phonemes with other places of articulation, such as [labial] or [dorsal]. As such, most of the previous ERP studies examining FUL have focused on place of articulation contrasts in German consonants and vowels (Eulitz and Lahiri, 2004; Scharinger and Lahiri, 2010; Cornell et al., 2011, 2013; Scharinger et al., 2012). While these studies provided support for [coronal] underspecification, few electrophysiological studies have tested [coronal] underspecification in English. Cummings et al. (2017) examined underspecification of /d/ and /b/, classified as [coronal] and [labial] respectively, in English-speaking adults. Each consonant was presented in a consonant-vowel (CV) combination (e.g., /bɑ/). Consistent with the predictions of FUL, the less specified /dɑ/ elicited a large MMN while no MMN was elicited by the more specified /bɑ/. Interestingly, not all participants demonstrated reliable mismatch responses. This suggested that [coronal] underspecification might not be a language universal phenomenon, at least as measured by the MMN (Scharinger et al., 2011).

Another way to test the language universal prediction of [coronal] underspecification is to examine the speech processing patterns of children. Thus, the primary goal of this study was to determine whether [coronal] underspecification occurs in young children. Using the same stimuli and stimulus presentation paradigm as Cummings et al. (2017), two early-acquired English consonants differing in place of articulation, [labial] /b/ and [coronal] /d/, were presented to 24 children (ages 4–6 years). If [coronal] underspecification is a language universal, it was predicted that the children would demonstrate asymmetrical response patterns similar to those of the adults in Cummings et al. (2017). That is, due to a place of articulation feature mismatch, the /dɑ/ deviant should elicit a large response when presented within the /bɑ/ standards, resulting in a large MMN. Alternatively, the /bɑ/ deviant would be a no-mismatch to the /dɑ/ standards, resulting in a much smaller MMN response. Such a result would indicate that young children have adult-like phonological representations, as suggested by the FUL.

An alternative, but not necessarily opposing, possibility is that /bɑ/ and /dɑ/ would elicit small and symmetrical MMN responses due to little response differences between the standards and deviants. Such a result would suggest that the two phonemes no-mismatch one another because there is no place of articulation feature contrast. This would be consistent with the predicted development of underspecification (Bernhardt, 1992). Thus, in this situation, [coronal] would still be the underspecified feature; however, the more marked [labial] place of articulation would not yet be correctly defined in the phonological representations. As a result, it could just be a matter of time before adult-like [coronal] underspecification patterns are present in children's responses.

In order to capture potential developmental trends and variations in phonological underspecification, children with typically developing *and* disordered phonological systems were included in the study. No previous study has used electrophysiological methods to examine speech processing in children with disordered phonological systems. Thus, a secondary goal of this study was to determine whether children with typically developing (TD) phonological systems and children with phonological disorders (PD) have distinctive speech processing neural signatures.

Phonological disorders are one subtype of speech sound disorders (McLeod and Baker, 2017). Children with speech sound disorders can demonstrate ‘any combination of difficulties with perception, articulation/motor production, and/or phonological representation of speech segments (consonants and vowels), phonotactics (syllable and word shapes), and prosody (lexical and grammatical tones, rhythm, stress, and intonation) that may impact speech intelligibility and acceptability' (International Expert Panel on Multilingual Children's Speech, 2012, p. 1). It has been claimed that the speech errors children with PD make may be due to knowledge deficits at the level of phonemic detail and/or at the level of phonemic contrasts (Rvachew and Jamieson, 1995). Specifically, speech sound perception problems may arise, at least in some cases, from faulty representation of the speech signal in the central auditory processing centers (Kraus, 2001). Children with PD may have difficulty creating phonological representations due to their inaccurate perception of speech sounds (Macken, 1980; Chaney, 1988; McGregor and Schwartz, 1992).

While stable perceptual representations of speech sounds would allow children to form detailed phonological representations that can be generalized across experiences, unstable neural encoding of speech could affect children's ability to process rapidly changing acoustic information that differentiates phonemes (Carr et al., 2016). This unstable neural encoding could lead to the creation of fuzzy (i.e., less detailed) phonological representations that do not allow children with PD to accurately discriminate between sounds that share similar articulatory features (e.g., voicing, articulatory placement, and/or articulatory manner). More specifically, if children with PD have fuzzy phonological representations, their ability to accurately discriminate one sound from another may be impaired. This suggests that if children do not have an appropriately detailed underlying phonological representation to access during speech production, speech production errors would likely occur. Thus, ultimately, children's speech production abilities might be highly dependent on their speech perception abilities (Scott, 2012).

While no published research has examined underlying perceptual mechanisms implicated in PD, studies involving children with developmental language disorders, reading disorders, and/or childhood apraxia of speech have identified atypical electrophysiological discriminatory responses to speech sounds (Kraus et al., 1996; Uwer et al., 2002; Sharma et al., 2006; Froud and Khamis-Dakwar, 2012; Volkmer and Schulte-Körne, 2018). With 18–25% of children with PD going on to develop reading difficulties or receive a dyslexia diagnosis (Cabbage et al., 2018), one potential deficit that children with PD might share with children with developmental language disorders and reading disabilities is an impairment in phonological processing or phonological representation (Elbro and Jensen, 2005; Boada and Pennington, 2006; Pennington and Bishop, 2009; Cabbage et al., 2018). It is plausible that the underlying neural responses representing the discrimination of speech sounds in children with PD could also be atypical in nature. Thus, it is likely that children with PD might have phonological representations that are different from those of TD children.

One possibility is that children with PD have developmentally immature phonological systems, possibly due to exceptionally sparse, or fuzzy, phonological representations. Evidence for this hypothesis could be provided by distinct mismatch response patterns found in the children with PD, as compared to the TD children. For example, in young children, stimulus mismatch responses are often either not a clear negativity or are fully positive in polarity (Cheour et al., 2002; Maurer et al., 2003). Positive mismatch responses (PMR) have been associated with neural development (Mueller et al., 2012). Specifically, different neural networks could be involved in the PMR and MMN, with superficial neural networks being recruited to produce the MMN while deep cortical neurons might generate the PMR (Ponton et al., 1999, 2002; Kral and Eggermont, 2007). PMR responses have often been reported in children who have or are at-risk-for dyslexia (Volkmer and Schulte-Körne, 2018). Given that dyslexia is phonologically based, these results suggest that children with poorer or less detailed phonological representations, such as children with PD, might demonstrate developmentally immature PMR to speech sounds. Thus, if there is a developmental trajectory of stimulus mismatch responses, children would first demonstrate a PMR that gradually shifts in polarity to eventually result in a large MMN. The transition from PMR to MMN might occur much earlier in TD children, as compared to children with impaired phonological processing.

## Methods

### Participants

Twenty-four children who were native speakers of (American) English participated in the study. Twelve of the children had typically developing (TD) phonological systems (9 male, 3 female; mean age: 5.8 years, range: 4.58–6.92 years). Twelve of the children had been previously diagnosed with a phonological disorder (PD) by a certified speech-language pathologist (6 male, 6 female; mean age 5.5 years, range: 4.00–6.92 years). All participants had normal vision and hearing within normal limits as determined by a standard audiometric screening and resided in a monolingual English-speaking household. The children with PD met the additional following criteria (Table 1): an oral-peripheral mechanism exam completed within normal limits (Robbins and Klee, 1987); speech articulation scores on the *Goldman-Fristoe Test of Articulation*−*3rd edition* (GFTA-3; Goldman and Fristoe, 2015) at least 1.25 standard deviations below the mean (standard scores of 80 or below), phonological process scores (i.e., speech error patterns) on the *Khan-Lewis Phonological Analysis*−*3rd edition* (KLPA-3; Khan and Lewis, 2015) at least 1.25 standard deviations below the mean (standard scores below 80), and a percent consonants correct (PCC) score (Shriberg and Kwiatkowski, 1982) for all the consonants on the GFTA-3 of 85% or less. Children with TD speech had standard scores of 90 or higher on the GFTA-3 and KLPA-3 and GFTA-3 PCC scores of 85% or higher.

**Table 1:** Characteristics of the typically developing (TD) children and children with phonological disorders (PD).

	**TD (*n* = 12)**	**PD (*n* = 12)**
Assessment age in years	5.81 (0.96)	5.51 (1.04)
	Range = 4.33–6.92	Range = 4.00–6.92
	*t*_(22)_ =0.732, *p >* 0.47	
GFTA−3: Standard score	107.17 (7.47)	65.75 (13.69)
	Range = 90–114	Range = 40–80
	*t*_(22)_ =9.202, *p <* 0.0001	
GFTA-3: Percent Consonants Correct (PCC)	96.34 (3.85)	70.84 (14.75)
	Range = 86–100	Range = 35–85
	*t*_(22)_ =5.795, *p <* 0.0001	
KLPA-3: Standard score	105.08 (7.22)	67.67 (9.28)
	Range = 90–112	Range = 50–79
	*t*_(22)_ =11.028, *p <* 0.0001	
Hearing	Within normal limits	Within normal limits

*Mean group scores and score range are presented. Standard deviations are reported within parentheses*.

*GFTA-3 refers to the standard score on the Goldman-Fristoe Test of Articulation 3rd Edition (Goldman and Fristoe, 2015). Standard scores between 85 and 115 are considered to be in the normal range*.

*KLPA-3 refers to the standard score on the Khan-Lewis Phonological Analysis 3rd Edition (Khan and Lewis, 2015). Standard scores between 85 and 115 are considered to be in the normal range*.

*Based on the Lifespan Reference Data for PCC (Austin and Shriberg, 1997), 5-year-old males should have an average PCC score of 86.9% while 5-year-old females should have an average PCC score of 87.3%*.

Regardless of their group assignment, all children correctly produced /b/ and /d/ in all the words on the GFTA-3 (i.e., four occurrences of /b/ and five occurrences of /d/). Thus, a behavioral speech perception task was not employed because all children were capable of accurately producing the study's phonemes of interest. This study was approved by the university institutional review board and a parent of each participant signed informed consent in accordance with the university human research protection program.

### Stimuli

The stimuli were the same as those used in Cummings et al. (2017) with adult participants; please refer there for more detailed information. Briefly, syllables (consonant + /ɑ/) were pronounced by a male North American English speaker. The average intensity of all the syllable stimuli was normalized to 65 dB SPL. Syllable duration was minimally modified (by shortening the vowel duration) so that all syllables were 375 ms in length. The vowel editing process began by identifying the most consistent, steady state portion (i.e., the middle) of the vowel. Then, a single sinusoid cycle of the vowel production was measured at 8 ms for /bɑ/ and 9 ms for /dɑ/. To achieve the target 375 ms for the entire syllable, 2 sinusoid cycles (i.e., 16 ms) were deleted in the /bɑ/ syllable and four cycles (i.e., 36 ms) were deleted in the /dɑ/ syllable. Each syllable token used in the study was correctly identified by at least 15 adult listeners.

Children heard two oddball stimulus sets, each containing the same four English speech consonant-vowel (CV) syllables: “ba” (/bɑ/), “da” (/dɑ/), “pa” (/pɑ/), and “ga” (/ɡɑ/). In one stimulus set, /bɑ/ served as the standard syllable, with the other three CV syllables serving as deviants. In the second stimulus set, /dɑ/ served as the standard syllable, with the other three syllables being deviants. Only responses to the /bɑ/ and /dɑ/ syllables will be addressed further since they served as both standard and deviant stimuli, which allowed for the creation of same-stimulus identity difference waves.

### Stimulus Presentation

The stimuli were presented in blocks containing 237 standard stimuli and 63 deviant stimuli (21 per deviant), with five blocks of each stimulus set being presented to each participant (i.e., 10 blocks total). Each block lasted ~6 min and the participants were given a break between blocks when necessary. Within the block, the four stimuli were presented using an oddball paradigm in which the three deviant stimuli (probability = 0.07 for each) were presented in a series of standard stimuli (probability = 0.79). Stimuli were presented in a pseudorandom sequence and the onset-to-onset inter-stimulus interval varied randomly between 600 and 800 ms. The syllables were delivered by stimulus presentation software (Presentation software, www.neurobs.com). The syllable sounds were played via two loudspeakers situated 30 degrees to the right and left from the midline 120 cm in front of a participant, which allowed the sounds to be perceived as emanating from the midline space. The participants sat in a sound-treated room and watched a silent cartoon video of their choice. The recording of the ERPs took ~1 h.

### EEG Recording and Averaging

Sixty-six channels of continuous EEG (DC-128 Hz) were recorded using an ActiveTwo data acquisition system (Biosemi, Inc., Amsterdam, Netherlands) at a sampling rate of 256 Hz. This system provides “active” EEG amplification at the scalp that substantially minimizes movement artifacts. The amplifier gain on this system is fixed, allowing ample input range (−264 to 264 mV) on a wide dynamic range (110 dB) Delta- Sigma (ΔΣ) 24-bit AD converter. Sixty-four channel scalp data were recorded using electrodes mounted in a stretchy cap according to the International 10–20 system. Two additional electrodes were placed on the right and left mastoids. Eye movements were monitored using FP1/FP2 (blinks) and F7/F8 channels (lateral movements, saccades). During data acquisition, all channels were referenced to the system's internal loop (CMS/DRL sensors located in the centro-parietal region), which drives the average potential of a subject (the Common Mode voltage) as close as possible to the Analog-Digital Converter reference voltage (the amplifier “zero”). The DC offsets were kept below 25 microvolts at all channels. Off-line, data were re-referenced to the common average of the 64 scalp electrode tracings.

Data processing followed an EEGLAB (Delorme and Makeig, 2004) processing pipeline. Briefly, data were high-pass filtered at 0.5 Hz using a pass-band filter. Line noise was removed using the CleanLine EEGLAB plugin. Bad channels were rejected using the trimOutlier EEGLAB plugin and the removed channels were interpolated. Source level contributions to channel EEG were decomposed using Adaptive Mixed Model Independent Component Analysis (AMICA) (Palmer et al., 2008) in EEGLAB (http://www.sccn.ucsd.edu/eeglab). Non-artifact, independent component (IC) scalp topographies were modeled as projections of single equivalent dipoles and clustered on the basis of dipole locations (Jung et al., 2000; Delorme and Makeig, 2004).

Epochs containing time points within a window encompassing 100 ms pre-auditory stimulus to 800 ms post-stimulus were baseline-corrected with respect to the pre-stimulus interval and averaged by stimulus type. On average, the individual data of TD children contained 763 (SD = 72) /bɑ/ standard syllable trials, 706 (SD = 162) /dɑ/ standard syllable trials, 85 (SD = 19) /bɑ/ deviant syllable trials, and 91 (SD = 9) /dɑ/ deviant syllable trials. On average, the individual data of children with PD contained 799 (SD = 172) /bɑ/ standard syllable trials, 895 (SD = 185) /dɑ/ standard syllable trials, 95 (SD = 17) /bɑ/ deviant syllable trials, and 96 (SD = 21) /dɑ/ deviant syllable trials.

### ERP and EEG Measurements

Three different data analysis strategies were used in the present study: (1) traditional mean amplitude repeated measure ANOVA analyses using averaged data, (2) cluster-based permutation analyses of averaged data (Bullmore et al., 1999; Groppe et al., 2011), and (3) general linear modeling of epoched (i.e., unaveraged) data (Pernet et al., 2011).

#### Mean Amplitude Measurements of Averaged Data

In an oddball paradigm, the MMN is typically examined by subtracting the standard ERP response from the deviant response in difference waves. The dual stimulus set nature of the present study allowed for the creation of “same-stimulus,” or identity, difference waveforms. These difference waves were created by subtracting the ERP response of a stimulus serving as the standard from that of the same stimulus serving as the deviant, across stimulus sets. For example, the ERP response for /bɑ/ as the standard was subtracted from the ERP response for /bɑ/ as the deviant (of the reversed stimulus set) (Eulitz and Lahiri, 2004; Cornell et al., 2011, 2013). The creation of identity difference waveforms eliminates the potential confound that variations in ERP morphology may result from acoustic stimulus differences, since the same stimulus is used to elicit both the standard and deviant responses.

Peak measurement of MMN was a multi-step process. While the MMN is typically maximal over fronto-central midline electrode sites (Näätänen et al., 1992), evidence of MMN-type activity was present in many electrodes. That observation, along with the fact that underspecification has not been examined in children before, nor has any prior ERP study examined phonological processing in children with PD, led to the inclusion of 28 electrodes in the analyses (F5/F6, F3/F4, F1/F2, Fz, FC5/FC6, FC3/FC4, FC1/FC2, FCz, C5/C6, C3/C4, C1/C2, Cz, CP5/CP6, CP3/CP4, CP1/CP2, and CPz)^1^. The most prominent mismatch response was observed in both groups 200–300 ms post-syllable onset. As this was consistent with the timing of the adult MMN response (Cummings et al., 2017), this time window was selected for data analysis. Phonological underspecification in the identity difference waves was analyzed using a Group (TD, PD) x Phoneme Type (/bɑ/, /dɑ/) x Anterior-Posterior (four levels) × Left-Right (seven levels) repeated measure ANOVA.

Given that the difference waves were generated from the standard and deviant syllable ERPs, the mean amplitude measurements of the standard and deviant waveforms were taken from the same 100 ms time as that of the MMN: 200–300 ms post-syllable onset. In terms of ERP waveform morphology, this measurement approximately captured the auditory N2. Phonological underspecification in these standard and deviant ERP waveforms was analyzed using a Group (TD, PD) x Phoneme Type (/bɑ/, /dɑ/) × Trial Type (Standard, Deviant) x Anterior-Posterior (4 levels) x Left-Right (seven levels) repeated measure ANOVA. Partial eta squared (η^2^) effect sizes are also reported for all significant effects and interactions. When applicable, Geiser-Greenhouse corrected *p*-values are reported.

#### Cluster Mass Permutation Tests of Averaged Data

The ERPs were submitted to repeated measures two-tailed cluster-based permutation tests (Bullmore et al., 1999; Groppe et al., 2011). These permutation test analyses provide better spatial and temporal resolution than conventional ANOVAs while maintaining weak control of the family-wise alpha level (i.e., it corrects for the large number of comparisons). To estimate the distribution of the null hypothesis, 2,500 permutations were used, which was more than twice the number recommended for a family-wise alpha level of 0.05 (Manly, 2006). These analyses enabled identification of differences between the underspecified /dɑ/ and the more specified /bɑ/. Thus, the high temporal resolution of this analysis could be used to identify a specific time period during which indices of underspecification were present.

Five different tests were conducted: (1) /bɑ/ vs. /dɑ/ identity MMN difference waveforms, (2) /bɑ/ vs. /dɑ/ standard ERPs, (3) /bɑ/ vs. /dɑ/ deviant ERPs, (4) /bɑ/ standard vs. /bɑ/ deviant ERPs, and (5) /dɑ/ standard vs. /dɑ/ deviant ERPs. These tests were conducted to identify group level (TD vs. PD) differences. The tests were also conducted separately for each group to examine phoneme type and/or trial type differences that were specific to TD children and/or children with PD. Each test included 28 different electrodes that encompassed four different anterior-posterior levels and seven different laterality measures: F5/F6, F3/F4, F1/F2, Fz, FC5/FC6, FC3/FC4, FC1/FC2, FCz, C5/C6, C3/C4, C1/C2, Cz, CP5/CP6, CP3/CP4, CP1/CP2, CPz. All of the time points (measured every 4 ms; 91 total time points) between 0 and 350 ms at the 28 scalp electrodes were included in the test (i.e., 2,548 total comparisons).

*T*-tests were performed for each comparison using the original data and 2,500 random within-participant permutations of the data. For each permutation, all t-scores corresponding to uncorrected *p*-values of 0.05 of less were formed into clusters. Electrodes within about 5.44 cm of one another were considered spatial neighbors, and adjacent time points were considered temporal neighbors. The sum of the t-scores in each cluster was the “mass” of that cluster. The most extreme cluster mass in each of the 2,501 sets of tests was recorded and used to estimate the distribution of the null hypothesis (i.e., no difference between conditions). The permutation cluster mass percentile ranking of each cluster from the observed data was used to derive *p*-values assigned to each member of the cluster. T-scores that were not included in a cluster were given a *p*-value of 1.

#### General Linear Modeling (GLM) of Epoched Data

GLM analyses were used to help account for the correlation in time and space dimensions found in EEG data, and to provide an alternate analysis technique to the repeated measure ANOVAs commonly used in ERP data analysis. They were also more robust to potential noise introduced by the trial number imbalance found in the standard and deviant syllable data generated by the oddball paradigm.

Following the protocol described in previous studies (Rousselet et al., 2011; Cummings et al., 2017), subjects' epoched data were modeled using LIMO EEG, an open source Matlab toolbox for hierarchical GLM, compatible with EEGLAB: https://gforge.dcn.ed.ac.uk/gf/project/limo_eeg/ (Pernet et al., 2011). The general linear model was used to examine single-trial ERP amplitudes, in microvolts, independently at each time point and each electrode. Parameters (β-values) were estimated at each electrode and time point independently, yielding a matrix of 64 (electrodes) x 103 (time points, from 0 to 400 ms post-stimulus in 4 ms steps) for each regressor. Similar electrode x time point matrices were computed for *R*^2^, F, and *p*-values for both the overall models and for each regressor (partial *F*-values). Probability values were determined using a permutation approach for which trial labels were permuted 1,000 times using a bootstrap-t technique (Wilcox, 2005). To examine underspecification of /dɑ/ as compared to /bɑ/ in standard and deviant trials, four GLM analyses of epoched data were conducted at the single-subject level: (1) /bɑ/ vs. /dɑ/ standards, (2) /bɑ/ vs. /dɑ/ deviants, (3) /bɑ/ standards vs. /bɑ/ deviants, and (4) /dɑ/ standards vs. /dɑ/ deviants.

##### Single subject GLM analyses

For each analysis, bootstrap paired *t*-tests were computed between the contrasts of interest at all time points across the entire scalp. Due to the small number of deviant trials and hence a low signal-to-noise ratio, most individual participants' analyses were not significant when controlled for multiple comparisons. Thus, for the purpose of examining phonological underspecification at a single-subject level, uncorrected data are reported.

To quantify and compare the individual results, two analyses were conducted. First, using the full-scalp uncorrected comparison analyses, the data of each participant were examined for a significant phoneme type or trial type difference (positive or negative) of at least 20 continuous milliseconds at electrode FCz during the 200–400 ms time window, which was the time window of the mismatch response observed in the grand averaged waveforms. The second analysis involved identifying a significant continuous 20 ms phoneme type or trial type difference in at least five separate electrode sites during the 200–400 ms time window; this analysis did not have to include electrode FCz, though this electrode was included in some cases.

## Results

**Figure 1:**
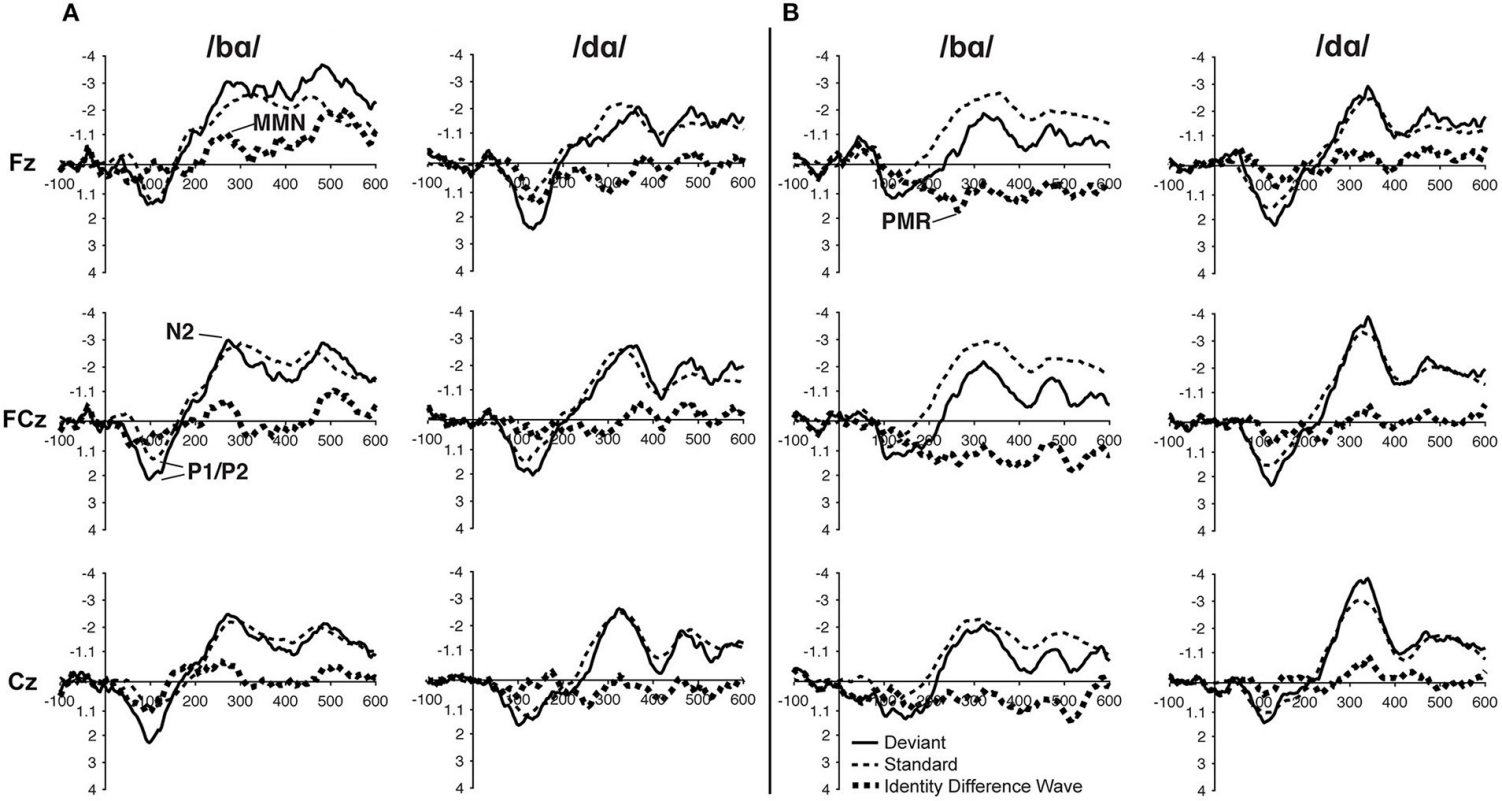
ERP waveforms elicited in the **(A)** typically developing (TD) children and **(B)** children with phonological disorders (PD). In each panel, the /bɑ/ syllable response is presented on the left and the /dɑ/ syllable response is on the right. The deviant waveforms represent the neural responses when the deviant syllable was presented within a stream of the opposite syllable standards. Subtracting the standard syllable response from the deviant syllable response resulted in the identity difference waves. Note that negative is plotted *up* in all waveforms.

In the ERP waveforms of both the TD children and children with PD, the standard and deviant /bɑ/ and /dɑ/ syllables elicited auditory P1/P2 at ca. 115 ms and auditory N2 at ca. 300 ms (Figure 1). In the same-stimulus identity difference waves of the TD children, an MMN response was observed at ca. 250 ms. Conversely, in the same-stimulus identity difference waves of the children with PD, a positive mismatch response (PMR) (Mueller et al., 2012) was evident at ca. 275 ms (Figure 1).

### ERP Mean Amplitude Results

#### Identity Difference Waves: MMN

**Figure 2:**
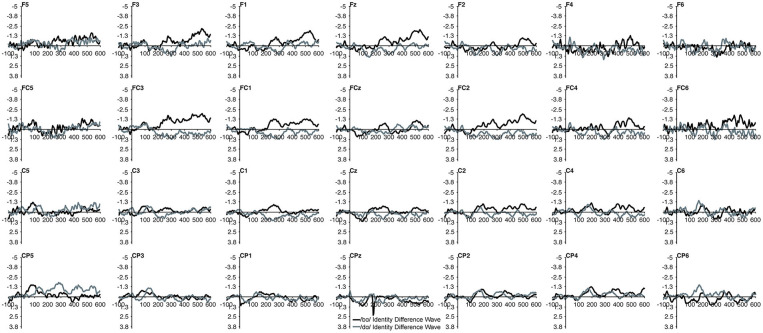
Identity difference waveforms of the TD children elicited by /bɑ/ and /dɑ/ across all 28 electrodes included in all analyses. The /bɑ/ responses are in black while the /dɑ/ responses are in gray.

**Figure 3:**
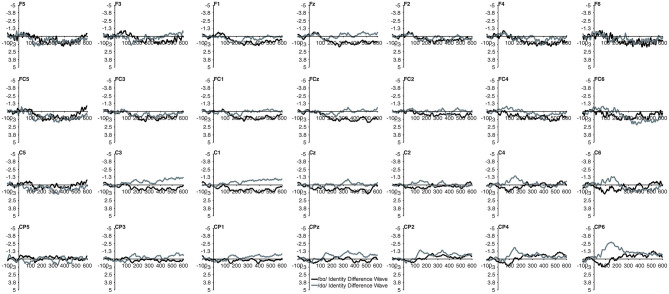
Identity difference waveforms of the children with PD elicited by /bɑ/ and /dɑ/ across all 28 electrodes included in all analyses. The /bɑ/ responses are in black while the /dɑ/ responses are in gray. Error bars represent SEM.

The MMN mean amplitude did not differ between the TD children and the children with PD (*p* > 0.28). For the combined groups, the mean amplitude of the /dɑ/ identity difference waveform was not significantly different from the /bɑ/ identity waveform (*p* > 0.94). A main effect of electrode location anteriority was found [*F*_(3, 66)_ = 4.894, *p* < 0.03, η^2^ = 0.182]. Mismatch responses tended to be more negative across posterior electrodes as compared to anterior electrodes, though pairwise comparisons revealed no significant differences. No main effect of left-right electrode location was observed (*p* > 0.91) (Figures 2, 3).

**Figure 4:**
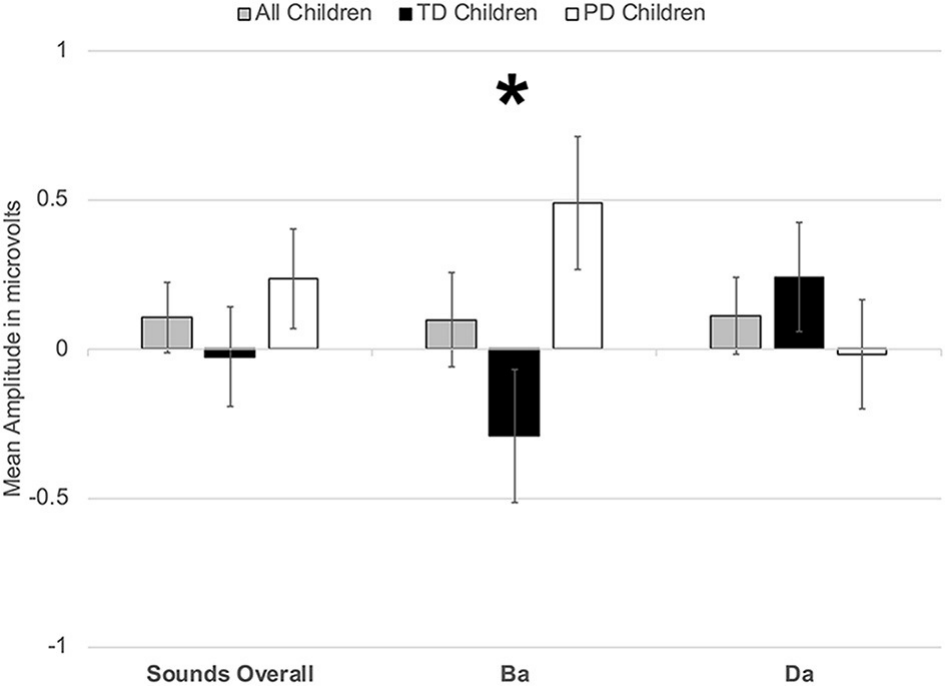
Average mean amplitudes for mismatch responses measured in identity difference waves from 200 to 300 ms post-syllable onset. Responses from the typically developing children are in black, responses from children with phonological disorders are in white, and combined responses across groups are in gray. Error bars represent SEM. The TD children demonstrated a negative mismatch response (MMN) to /bɑ/ while the children with PD demonstrated a positive mismatch response (PMR). The The * symbol represents a significant difference.

However, a significant group x phoneme type interaction was observed [*F*_(1, 22)_ = 9.817, *p* < 0.006, η^2^ = 0.309] (Figure 4). When examining just the responses elicited by /bɑ/, the TD children had a negative response (i.e., MMN) while the children with PD demonstrated a large positive mismatch response (i.e., PMR) [*F*_(1, 22)_ = 6.126, *p* < 0.03, η^2^ = 0.218] (Figures 4, 8B); significant group differences were not observed in the /dɑ/ mismatch responses (*p* > 0.32). The TD children's mismatch responses to /bɑ/ and /dɑ/ did not significantly differ (*p* > 0.08) (Figures 4, 9A). The /bɑ/ mismatch response of the children with PD was significantly more positive than was the mismatch response to /dɑ/ [*F*_(1, 11)_ = 7.744, *p* < 0.02, η^2^ = 0.413] (Figures 4, 9D). Thus, this interaction was primarily driven by the opposing directions of the group mismatch responses elicited by /bɑ/.

#### Standard and Deviant Waveforms

**Figure 5:**
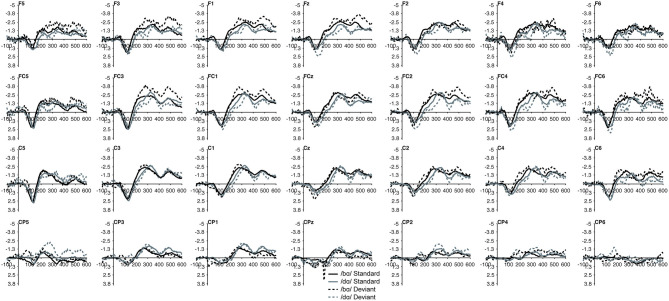
Standard and deviant waveforms of the TD children elicited by /bɑ/ and /dɑ/ across all 28 electrodes included in all analyses. The /bɑ/ responses are in black while the /dɑ/ responses are in gray. Standard responses are solid lines while deviant response are dashed lines.

**Figure 6:**
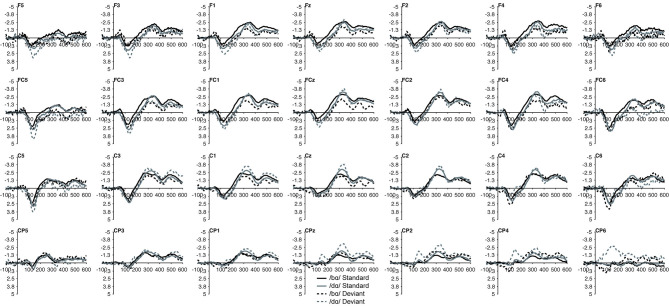
Standard and deviant waveforms of the children with PD elicited by /bɑ/ and /dɑ/ across all 28 electrodes included in all analyses. The /bɑ/ responses are in black while the /dɑ/ responses are in gray. Standard responses are solid lines while deviant response are dashed lines.

No group main effect was found (*p* > 0.30); overall, the ERP mean amplitudes of the TD children and the children with PD did not differ. A main effect of phoneme type was found, as across both groups, the ERP mean amplitude of the /bɑ/ waveform was significantly more negative (i.e., larger) than that of /dɑ/ [*F*_(1, 22)_ = 25.049, *p* < 0.0001, η^2^ = 0.532]. No main effect of trial type was observed (*p* > 0.37); the standard and deviant waveforms did not differ from each other. Across both groups, a main effect of left-right [*F*_(6, 132)_ = 4.341, *p* < 0.02, η^2^ = 0.165] electrode location was found. Significantly smaller responses were recorded over the most lateral electrodes (Far Left/Far Right), as compared with their nearest neighbors (Mid Left/Mid Right; all *p* < 0.02); no other electrode left-right effects were significant. No effect of anterior-posterior electrode location was observed (*p* > 0.17) (Figures 5, 6).

**Figure 7:**
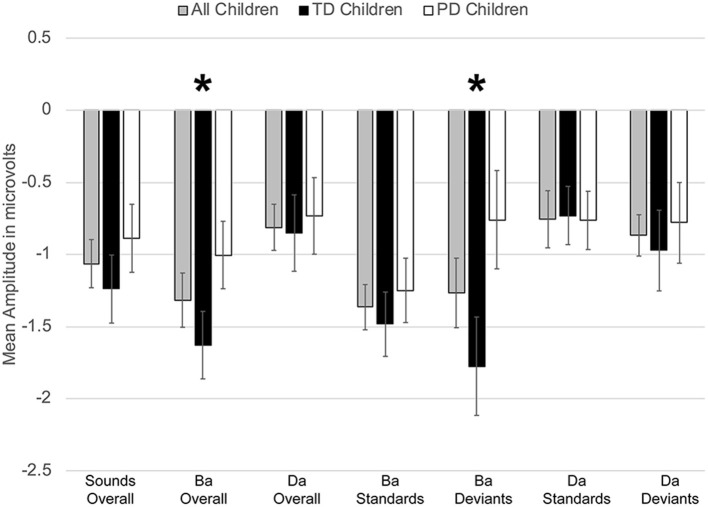
Average mean amplitudes for standard and deviant ERPs from 200 to 300 ms post-syllable onset. Responses from the typically developing children are in black, responses from children with phonological disorders are in white, and combined responses across groups are in gray. Error bars represent SEM. The ERP responses elicited by the /bɑ/ deviants in the TD children were significantly larger (i.e., more negative) than those of the children with PD. The * symbol represents a significant difference.

**Figure 8:**
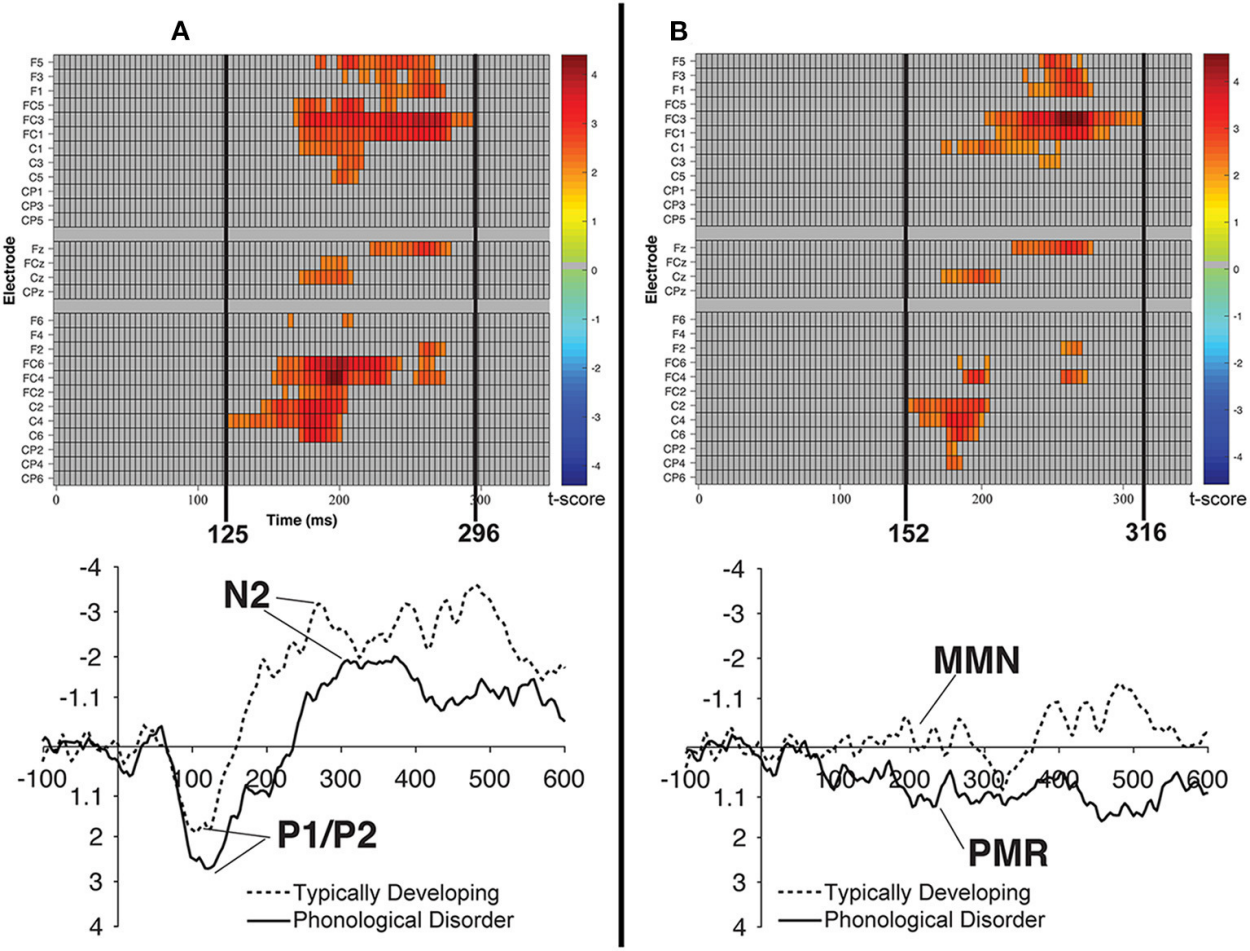
Raster diagrams and waveforms illustrating group (TD vs. PD) differences in the processing of the /bɑ/ stimuli. On the left **(A)** are group differences in response to the /bɑ/ deviant. TD children demonstrated a more negative response than did children with PD. On the right **(B)** are group differences in response to the /bɑ/ identity difference wave. TD children demonstrated a negative mismatch response while children with PD demonstrated a positive mismatch response. For the raster diagrams, colored rectangles indicate electrodes/time points in which the ERPs to one stimulus are significantly different from those to another. The color scale dictates the size of the *t*-test result, with dark red and blue colors being more significant. Gray areas indicate electrodes/time points at which no significant differences were found. Note that the electrodes are organized along the y-axis somewhat topographically. Electrodes on the left and right sides of the head are grouped on the figure's top and bottom, respectively; midline electrodes are shown in the middle. Within those three groupings, y-axis top-to-bottom corresponds to scalp anterior-to-posterior.

On the other hand, a group x phoneme type interaction was observed [*F*_(1, 22)_ = 7.347, *p* < 0.02, η^2^ = 0.250] (Figure 7). While the TD children's ERP responses to /bɑ/ were significantly more negative than their responses to /dɑ/ [*F*_(1, 11)_ = 59.771, *p* < 0.0001, η^2^ = 0.845], no phoneme type differences were observed in the children with PD (*p* > 0.21). A group x phoneme type x trial type interaction was also found [*F*_(1, 22)_ = 9.817, *p* < 0.006, η^2^ = 0.309]. Significant group differences were not observed when responses to the /bɑ/ standards (*p* > 0.46), /dɑ/ standards (*p* > 0.47), and /dɑ/ deviants (*p* > 0.89) were examined separately. However, the ERP responses elicited by the /bɑ/ deviants in the TD children were significantly larger than those of the children with PD [*F*_(1, 22)_ = 4.442, *p* < 0.05, η^2^ = 0.168] (Figures 7, 8A). Thus, phonological underspecification group differences were most prevalent in the responses elicited by the /bɑ/ deviants.

### Cluster Permutation Analysis Results

#### Identity Difference Waveforms

##### TD vs. PD identity difference waves

Cluster-level mass permutation procedures encompassing the timeline of the P1/P2, N2, and MMN (0–350 ms) were applied to the data. Figure 8B shows one cluster extending from 152 to 316 ms that signified the time period during which the /bɑ/ difference waves of the TD children differed from those of the children with PD; the smallest significant t-score was: *t*_(23)_ = 2.07, *p* < 0.05. This effect was driven by essentially opposite mismatch responses, with an MMN response present in the difference waves of the TD children, while a PMR was present in the difference waves of the children with PD. No significant clusters were identified when examining group differences in the /dɑ/ identity difference wave responses.

##### TD identity difference waves: /bɑ/ vs /dɑ/

No significant clusters were identified when examining the difference between the /bɑ/ and /dɑ/ identity difference waves.

##### PD identity difference waves: /bɑ/ vs /dɑ/

**Figure 9:**
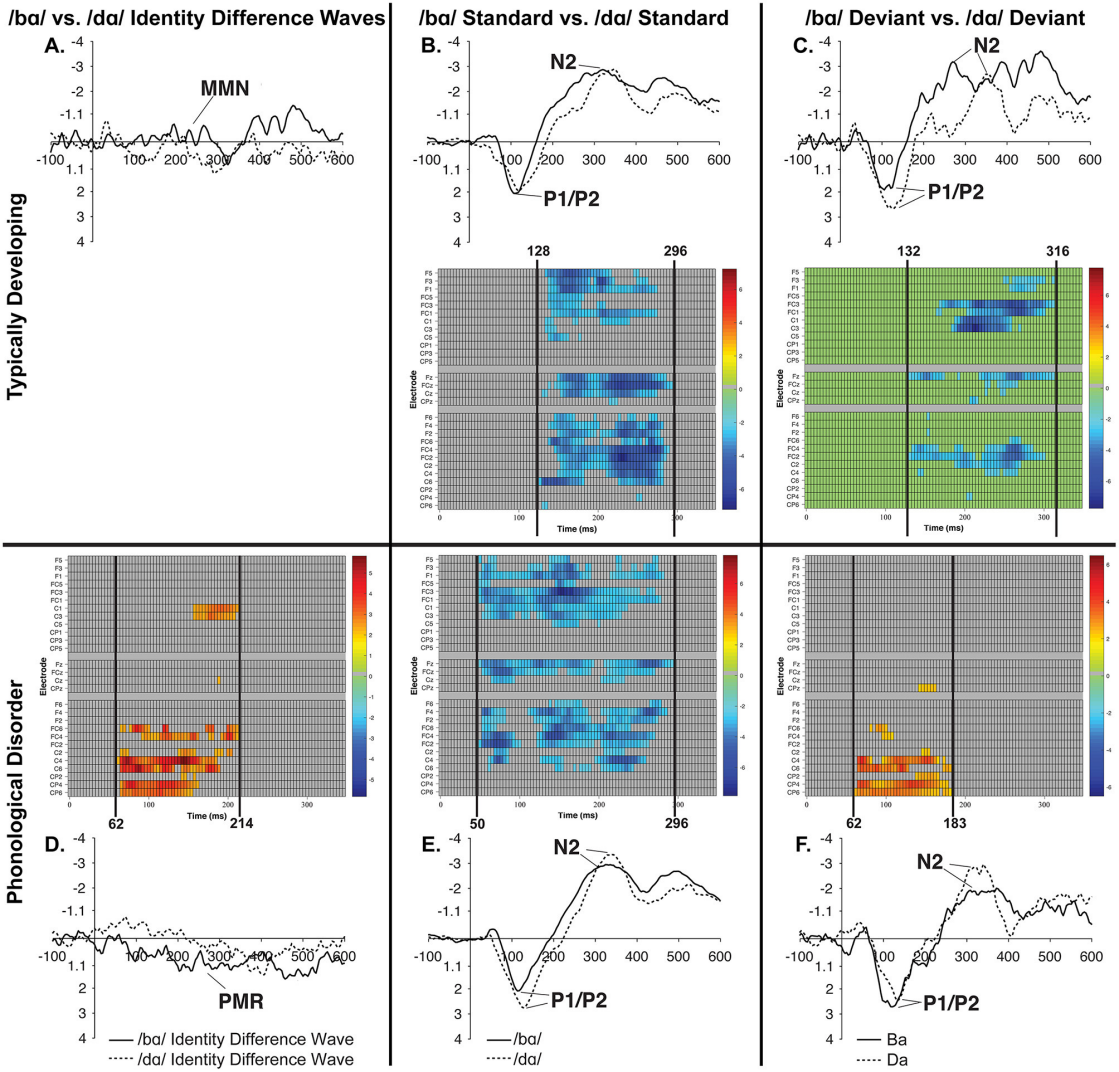
Raster diagrams and waveforms illustrating /bɑ/ and /dɑ/ processing differences in the TD children and children with PD. Responses from TD children are on the top **(A–C)**, while responses from children with PD are on the bottom **(D–F)**. In the left column are /bɑ/ and /dɑ/ identity difference wave comparisons **(A,D)**, the middle column contrasts /bɑ/ and /dɑ/ standards **(B,E)**, and the right column contrasts /bɑ/ and /dɑ/ deviants **(C,F)**. Both groups of children demonstrated similar processing patterns in the standard and deviant analyses, as the /bɑ/ elicited more negative responses than did the /dɑ/. The /bɑ/ mismatch response of the children with PD was significantly more positive than the mismatch response to /dɑ/, especially over the right hemisphere electrodes; no significant differences were observed in the identity difference waves of the TD children.

A predominantly right hemisphere cluster extending from 62 to 214 ms signified the time period during which the /dɑ/ difference wave differed from the /bɑ/ difference wave; the smallest significant t-score was: *t*_(11)_ = 2.208, *p* < 0.007 (Figure 9D). During this time period, a PMR response was seen in the /bɑ/ difference wave while no significant mismatch response was evident in the /dɑ/ difference wave.

#### Standard and Deviant Waveforms

##### TD vs. PD standard and deviant waveforms

Consistent with the mean amplitude measurements, the group comparison of /bɑ/ deviant responses yielded a significant cluster. A broadly distributed effect from 125 to 296 ms signified the time period during which the /bɑ/ deviants of the TD children differed from those of the children with PD; the smallest significant t-score was: *t*_(22)_ = 2.075, *p* < 0.02 (Figure 8A). Since this time window primarily encompassed the time period between the peak of the auditory P1/P2 and the peak of the auditory N2 ERP responses, this difference implies that the /bɑ/ deviant response elicited in the TD children was significantly more negative (i.e., larger N2) than that of the children with PD. No other significant clusters were identified in the group comparisons.

##### TD standard and deviant waveforms

No significant clusters were identified when examining the difference between the /bɑ/ standards and /bɑ/ deviants, or the difference between /dɑ/ standards and /dɑ/ deviants. Thus, neither phoneme type elicited a mismatch response wherein the deviant stimulus was reliably larger (or smaller) than the corresponding standard stimulus.

When contrasting the phoneme differences, a broadly distributed effect from 128 to 296 ms signified the time period during which the /dɑ/ standards differed from the /bɑ/ standards; the smallest significant t-score was: *t*_(11)_ = −2.02, *p* < 0.001 (Figure 9B). This difference implies that the /bɑ/ standards elicited more negative (i.e., larger N2) responses than did the /dɑ/ standards.

Similarly, when the /bɑ/ and /dɑ/ deviant syllables were contrasted, a significant effect from 132 to 316 ms signified the time period during which the /bɑ/ deviants elicited more negative (i.e., larger N2) responses than the /dɑ/ deviants; the smallest significant t-score was: *t*_(11)_ = −2.211, *p* < 0.002 (Figure 9C). This effect was more localized to the fronto-central and central electrode locations.

##### PD standard and deviant waveforms

No significant clusters were identified when examining the difference between the /bɑ/ standards and /bɑ/ deviants, or the difference between /dɑ/ standards and /dɑ/ deviants. A broadly distributed effect from 50 to 296 ms signified the time period during which the /bɑ/ standards differed from the /dɑ/ standards; the smallest significant t-score was: *t*_(11)_ = −2.204, *p* < 0.002 (Figure 9E). Similarly, when the /bɑ/ and /dɑ/ deviant syllables were contrasted, a significant right hemisphere localized cluster was present from 62 to 183 ms; the smallest significant t-score was: *t*_(11)_ = 2.20, *p* < 0.04 (Figure 9F).

### Single-Trial GLM Analyses

The single-subject data show the prevalence of potential [coronal] underspecification in English-speaking children. Individual participants' full scalp analyses of the four comparisons are presented in the online Supplementary Material. While the vast majority of the participants' individual significant effects occurred during the time period of the mismatch response (~200–400 ms post-syllable onset), some effects were observed both before and after this time window. Table 2 provides an overview of the single-subject findings.

**Table 2:** Single-subject results from the four LIMO GLM analyses.

**Child**	**Age**	**Group**	**/b**ɑ**/ Standards vs. /b**ɑ**/ Deviants**		**/d**ɑ**/ Standards vs. /d**ɑ**/ Deviants**		**/b**ɑ**/ Standards vs. /d**ɑ**/ Standards**		**/b**ɑ**/ Deviants vs. /d**ɑ**/ Deviants**	
			**FCz**	**5 elect**.	**FCz**	**5 elect**.	**FCz**	**5 elect**.	**FCz**	**5 elect**.
1	5.00	TD			X	X	X	X	X	X
2	6.33	TD	X	X				X		X
3	6.33	TD		X	X	X	X	X		X
4	6.92	TD		X				X		X
5	6.67	TD				X	X	X		
6	5.00	TD		X		X	X	X		X
7	4.33	TD		X		X	X	X		X
8	6.17	TD	X	X		X		X	X	X
9	4.92	TD				X		X		
10	6.67	TD				X	X	X	X	X
11	6.75	TD	X			X		X		X
12	4.58	TD		X		X		X		X
13	6.92	PD		X		X		X		X
14	5.33	PD		X		X				
15	5.50	PD				X		X		
16	4.00	PD			X	X		X		X
17	5.08	PD	X	X	X	X		X		X
18	5.83	PD	X	X				X		X
19	6.33	PD		X		X		X		
20	4.83	PD	X	X		X	X	X	X	X
21	6.83	PD		X		X		X		X
22	5.17	PD		X		X		X		X
23	4.00	PD	X	X	X	X		X	X	X
24	6.58	PD						X		X

*Each participant's data were examined for a significant (though uncorrected for multiple comparisons) phoneme type or trial type difference of at least 20 continuous milliseconds at electrode FCz, and across at least five separate electrode sites (that did not have to include FCz), between 200 and 400 ms post-syllable onset—the time window of the mismatch response. An “X” identifies participants who showed a significant effect for each measurement*.

#### TD Children

In the /bɑ/ standard vs. deviant analysis, 3/12 participants demonstrated a significant difference between the trial types at FCz, with 7/12 participants demonstrating a significant difference elsewhere across at least five electrodes. In the /dɑ/ standard vs. deviant analysis, 2/12 participants had a significant difference between trial types at FCz, though 10/12 participants demonstrated a significant trial type difference elsewhere. Thus, more TD children demonstrated some evidence of a /dɑ/ mismatch response than a /bɑ/ mismatch response. Indeed, five children only demonstrated distributed mismatch responses to /dɑ/, while two children only demonstrated responses to /bɑ/.

When the standards of the two syllables were compared, ERP activities in 6/12 participants differentiated /bɑ/ and /dɑ/ at FCz, and all 12 participants demonstrating a sensitivity to phoneme type across other scalp locations. The differences between the /bɑ/ and /dɑ/ deviants were not quite as prevalent, as 3/12 participants demonstrated a phoneme type difference at FCz, and 10/12 participants showed a phoneme type difference at other electrode sites.

#### Children With PD

In the /bɑ/ standard vs. deviant analysis, 4/12 participants demonstrated a significant difference between the trial types at FCz, with 9/12 participants demonstrating a significant trial type difference elsewhere across at least five electrodes. In the /dɑ/ standard vs. deviant analysis, 3/12 participants showed a significant trial type difference at FCz and 10/12 participants demonstrated a significant trial type difference elsewhere. Thus, evidence of mismatch responses to both /bɑ/ and /dɑ/ was present in the children with PD. Two children only demonstrated mismatch responses to /dɑ/, one child only demonstrated a mismatch response to /bɑ/, and one child did not demonstrate a response to either syllable.

When the standards of the two syllables were compared, 1/12 participants demonstrated a significant difference between /bɑ/ and /dɑ/ at FCz, while 11/12 participants demonstrated a phoneme type difference across other scalp locations. The differences between the /bɑ/ and /dɑ/ deviants were less clear, as 2/12 participants demonstrated a significant phoneme type difference at FCz, and 9/12 participants showed a phoneme type difference at other electrode sites.

## Discussion

This study examined phonological underspecification in 4- to 6-year-old children with typically developing (TD) and disordered (PD) phonological systems. Two phonemes, [labial] /b/ and [coronal] /d/, were presented to children within consonant-vowel syllables. In the TD children, no asymmetrical MMN responses were elicited by the underspecified /dɑ/ and specified /bɑ/; children's mismatch responses were equivocal. On the other hand, in the children with PD, the /bɑ/ mismatch response was significantly more positive than was the mismatch response to /dɑ/. Both of these findings were contrary to previous to adult [coronal] underspecification findings (Cummings et al., 2017) wherein the underspecified /dɑ/ elicited a larger MMN than did the specified /bɑ/.

Analysis of single-subject responses via GLM did not reveal any predictable underspecification patterns. Overall, more participants demonstrated a measurable /dɑ/ mismatch response as compared to /bɑ/. However, there was variability, as some children had a /bɑ/ mismatch response, but no /dɑ/ mismatch response. That so many children did not demonstrate mismatch asymmetries in their processing of /bɑ/ and /dɑ/ provides strong evidence against the language universality of [coronal] underspecification. Or, at the very least, the predictions should be modified to consider how [coronal] underspecification might develop in children.

### A Developmental Model of Phonological Underspecification

**Figure 10:**
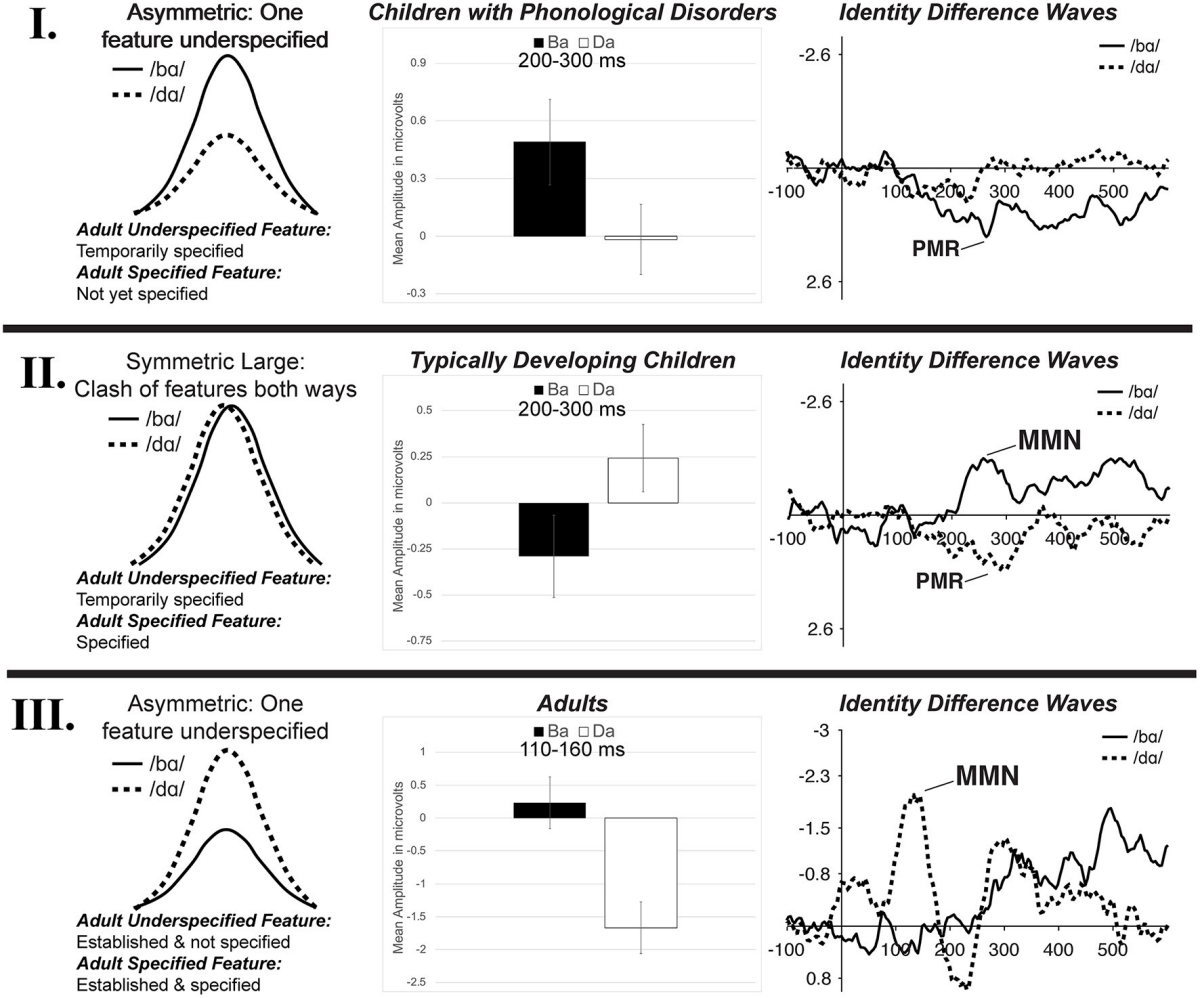
The three stages of the proposed developmental model of phonological underspecification. Children with PD are hypothesized to be in **Stage I**, TD children are hypothesized to be in **Stage II**, while adults are presumed to be in **Stage III**. The left column represents a schematic representation of possible mismatch responses in each stage (based on Schluter et al., 2016). The proposed development and specification of features is listed, with /d/ presumed to be the underspecified phoneme and /b/ the specified phoneme. The middle column displays the mean amplitude of the mismatch responses found in the /bɑ/ and /dɑ/ identity difference waves of each population during the mismatch analysis window. The right column presents the identity differences waves of each population from electrode FC1. See text for more detail.

Since children did not demonstrate the FUL-predicted adult MMN asymmetry pattern, it is likely that underspecification develops over time. It is proposed that there is a developmental trajectory for phonological underspecification, and the TD children and children with PD were functioning at different points on this continuum. Schluter et al. (2016) predicted multiple types of potential underspecification MMN responses depending on the type of feature specification and the manner in which the phonological system characterized sounds. This type of framework has been adopted here to represent three potential stages in the development of phonological underspecification based on the current data from children with PD (possibly representing an earlier stage of development), children with TD, and adults. That is, the data suggest a developmental model of phonological underspecification (Figure 10).

Within the proposed developmental model, the first stage of phonological underspecification occurs when the child is developing and defining the phonological representation of the underspecified phoneme, /d/ (Figure 10—**I**). Since underspecified phonemes are considered to be less complex than specified phonemes, it is proposed that the features of the underspecified phonemes are developed prior to those of the specified phonemes. Thus, in this stage, the features of the specified phoneme may not yet be defined. In the case of /b/ and /d/, [coronal] is defined and temporarily specified within the /d/ representation, while the [labial] place of articulation feature is not yet a part of the /b/ phonological representation. This means that when the underspecified phoneme, /d/, is the standard, it sets up a [coronal] feature expectation. When /b/ is the deviant, its representation does not contain this feature; as such, this feature contrast would be considered a mismatch. Alternatively, when the /b/ is the standard, it does not specify a place of articulation feature. As a result, even though /d/ had a contrasting feature, this contrast would be considered a no-mismatch, resulting in a small or no mismatch response. In the present study, the children with PD demonstrated this response pattern, as a positive mismatch response was evident in the /bɑ/ identity difference waves, while no mismatch response was present in the /dɑ/ difference waves.

The model proposes that the second stage of phonological underspecification occurs when the child develops and defines the phonological representation of the more specified phoneme (Figure 10—**II**). The features of the more complex specified phoneme, /b/, are now phonologically defined in the phonological representation, while the features of the underspecified phoneme continue to be temporarily specified. Thus, in the case of /b/ and /d/, the [labial] and [coronal] places of articulation have now been phonologically assigned to their respective phonemes. With both phonemes having fully specified phonological representations, specific features are expected in both oddball conditions. When the /b/ is the standard, [labial] is expected; when /d/ is the standard, [coronal] is expected. Thus, when either phoneme is the deviant stimulus, the feature contrast would be a mismatch. As a result, both the specified and underspecified phonemes are predicted to elicit mismatch responses—no asymmetrical response pattern should occur. The TD children in the present study demonstrated this response pattern, as mismatch responses were present in both the /bɑ/ and /dɑ/ identity difference waves.

The final stage of the proposed developmental model of phonological underspecification occurs when only the features of the specified phoneme are defined in the phonological representation, while the underspecified phoneme's features are no longer specified, but considered to be the default (Figure 10—**III**). This stage assumes that over time children learn which phoneme features are phonologically defined within the representation, and which ones are default features. In this stage, [coronal] is assumed to be the default place of articulation unless other evidence is provided. This means that [labial] is specified for /b/, while /d/ does not have a specified place of articulation. In terms of the oddball paradigm, the specified phoneme then sets up a specific feature expectation, which in this case is [labial]. When the underspecified phoneme, /d/, is the deviant, the feature contrast results in a mismatch. Alternatively, when the underspecified phoneme is the standard, its lack of a place of articulation creates no feature expectation. As a result, even though the specified phoneme has a contrasting feature, a no-mismatch occurs when it is the deviant—resulting in a small or no mismatch response. The adults in Cummings et al. (2017) followed this response pattern.

This proposed developmental model is consistent with previous proposals of children's speech processing (Bernhardt, 1992; Kuhl, 2000). Recall that Bernhardt (1992) proposed that children first develop and define underspecified features and then gradually add more specified features. This claim is supported by the data of the children with PD. The children with PD appeared to develop the underspecified [coronal] feature prior to that of the more specified [labial]. That is, while children had phonologically assigned the [coronal] feature to /d/, [labial] was not yet defined in the phonological representation of /b/. This suggests that children have access to more phonological information about /d/ than /b/ early in development. This acquisition pattern could be the explanation for why the children with PD demonstrated exactly opposite MMN patterns as those of adults. That is, the children had the [coronal] feature defined in their phonological representations while adults' representations contained [labial].

Moreover, data from the TD children suggest that children first develop and phonologically define all the features of /b/ and /d/ prior to refining their phonological representations through the elimination of the redundant default [coronal] feature. This phonological organization of the /b/ and /d/ representations likely resulted in mismatch responses for both phonemes. These findings suggest that the children had not yet learned that certain default underspecified features, such as [coronal], do not need to be assigned to phonological representations. Thus, the composition of phonological representations appears to develop and change over time.

In sum, the proposed developmental model of phonological underspecification is meant to provide a framework within which to examine the universality of the theory in children. The particular predictions of underspecification will need to be extensively tested in developmental populations to provide converging, or diverging, evidence for the proposal put forth here. Longitudinal and/or cross-sectional studies could clarify how phonological representations are established in development.

### A Developmental Trajectory for Mismatch Responses

Two types of mismatch responses, negative (MMN) and positive (PMR), were observed in the present study. In the TD children, an MMN was present in the /bɑ/ identity difference waves while a PMR was present in the /dɑ/ difference waves. Moreover, a PMR was observed in the /bɑ/ difference waves of the children with PD. While the present study considered both the MMN and PMR as characterizing mismatch responses within the FUL framework, the polarity differences of the mismatch responses suggest a developmental trajectory, with children first demonstrating a PMR that gradually shifts in polarity to an MMN.

The polarity differences in the mismatch response provide additional evidence of a developmental trajectory of phonological underspecification in children. That is, feature contrasts that are acquired first elicit the PMR. Once the features are more established, the feature contrasts elicit the MMN. For example, the children with PD only demonstrated a PMR, suggesting early-developing phonological knowledge pertaining to just the less complex, underspecified phoneme. Conversely, the TD children demonstrated both positive and negative mismatch responses. The MMN was associated with the underspecified phoneme and the PMR was associated with the specified phoneme. In this situation, the TD children's knowledge of the underspecified phoneme was more extensive, leading to the MMN. Thus, they may have still been in the process of developing and defining the features of the more specified phoneme, resulting in the PMR. Finally, the more specified phoneme elicited the MMN in adults (Cummings et al., 2017). It is assumed that the adults had adequate and extensive knowledge of the phoneme, which elicited the negative mismatch response. Thus, the longer a feature had been assigned phonological meaning and defined within a phonological representation, the more negative its mismatch response. Mismatch polarity could characterize the depth and detail of phonological knowledge.

### Typically Developing vs. Disordered Phonological Systems

As discussed above, the TD children and children with PD were likely following the same developmental trajectory of phonological underspecification, but were functioning at different stages. That is, the TD children demonstrated developmentally more mature responses than the children with PD, based on the pattern and polarity of their mismatch responses. Thus, children with PD do not appear to have the same extent of phonological knowledge as their same-aged, TD peers. This finding is consistent with previous evidence that language disordered populations demonstrate different mismatch responses as compared to their TD peers. For example, as compared to their TD peers, PMR responses have often been reported in children who have or are at-risk-for dyslexia (Volkmer and Schulte-Körne, 2018).

Overall, the processing differences in the two groups primarily centered around the more specified sound, /b/. Based on their mismatch response pattern, it would appear that the children with PD did not have the specified place of articulation feature [labial] sufficiently developed and defined within their phonological representations while the TD children did. Alternatively, the children with PD did not differ from the TD children in their standard and deviant ERP responses to /dɑ/. This finding suggests that there is an early processing advantage for less specified phonemes. Even if default features are initially stored in the phonological representations, those features may be easier to acquire than specified features.

This study provides evidence that the entire phonological system is not impaired in children with PD, as the underspecified /d/ phoneme elicited neural responses similar to those of TD children. This may be surprising, given their significant differences on the standardized speech articulation and phonological assessments. If children produce speech by accessing their underlying phonological representations, it would be assumed that children with large numbers of speech production errors have incorrect representations. However, it is important to remember that the children in both groups could accurately produce /d/, as evidenced by their test scores. Thus, it is possible that the phonological representations of /d/ were similar in both groups of children. Conversely, the data suggest that the representations of /b/ were not the same in the two groups of children, even though the children could also accurately produce that phoneme. As no previous study has examined the neural indices of speech processing in children with PD, it is presently unknown how children with PD would respond to phonemes they could not produce correctly. It is possible that an incorrect or extremely sparse phonological representation of affected phonemes is an underlying mechanism of the speech production errors observed in children with PD.

This work also has important potential clinical implications. It is nearly impossible for speech-language pathologists (SLPs) to predict how well a child might perform in treatment, as outcomes are varied. While behavioral predictors of speech treatment outcomes have not yet been identified, it is possible that the neural patterns children demonstrate prior to beginning treatment might indicate how well they will learn to produce a treated sound. That is, ERP responses could be indices of children's speech production ability and/or speech treatment effectiveness. Specifically, the present evidence suggests that children with PD might be recruiting developmentally immature neural networks for the processing of speech sounds, which might not allow for full and accurate processing and discrimination of phonological information. SLPs could use such information to design intervention programs that target not only speech production, but general phonological knowledge and/or speech perception skills, which could lead to better overall intervention outcomes.

### Limitations

The present study was the first to examine neural indices of phonological underspecification in children, both with typically developing and disordered phonological systems. Specifically, the present study focused on preschool-aged children between 4- and 6-years of age, as that is the age in which the highest percentage of children are diagnosed with PD (Shriberg et al., 1999; Law et al., 2000). As such, it is likely we missed the earliest stages of phonological development whose precursors are present in the infant speech perception work (Kuhl, 2000; Werker and Hensch, 2015). It would be useful to examine underspecification in younger children to see if TD children demonstrate Stage I at an earlier age than children with PD, and to see if there could be an even earlier stage that we could not identify with our present population and age groups. Moreover, examining underspecification in older children who have theoretically acquired all of their speech sounds (McLeod and Crowe, 2018; Crowe and McLeod, 2020) could provide information about how and when adult-like phonological knowledge is acquired.

Individual differences within and across groups are an inherent confounds when working with children in general, and disordered populations in particular. For example, while the TD children's mismatch responses to /bɑ/ and /dɑ/ did not reliably differ, a strong trend was observed. That this trend did not reach significance suggests that there might have been some variability in the TD sample. Moreover, while all children met the basic criterion to be included in one group or the other, there was still a range of severity of speech production difficulty in the children with PD. The single-trial analyses (Supplementary Figures 1–6) show that participants demonstrated a wide range of responses to the stimuli. It is possible that there are subtypes of PD, and different neural response patterns could be used to identify them. However, much larger groups of children would be necessary to address this issue. Future studies with new and/or larger groups of participants can provide converging evidence for the underspecification evidence provided here.

Identity difference waves were included to control for basic differences in acoustic detail present in the /bɑ/ and /dɑ/ stimuli. Still, it is still possible that the physical acoustic differences of /bɑ/ and /dɑ/ alone were responsible for the observed MMN response differences (Näätänen et al., 2007). However, from a sonority standpoint (Clements, 1990), the sonority difference between /b/ and /ɑ/ is acoustically the same as that of /d/ and /ɑ/. Thus, neither consonant establishes a stronger syllable onset than the other; they are acoustically functioning at a similar level. The frequency of occurrence, or phonotactic probability, of phoneme combinations could have also affected the MMN responses (Bonte et al., 2005; Näätänen et al., 2007). However, the phonotactic probability (Vitevitch and Luce, 2004) of the single phonemes /b/ and /d/ were nearly identical (0.0512 and 0.0518, respectively) and the phonotactic probability of the syllables were quite similar (0.0039 and 0.0023, respectively). Thus, it does not seem that the frequency with which children encounter the phonemes and syllable combinations in the ambient language were driving the response differences.

It is possible that the general acoustic perceptual ability was different in the two groups of children. For example, while the left inferior frontal gyrus (IFG) has been associated with articulatory-based speech codes (Poeppel et al., 2008), it has been suggested that atypical right hemisphere (i.e., IFG) processing may impact phonological processing (Goswami, 2011). When looking at the PD children's responses in Figure 9, many of the phoneme response differences in Panels A and C are only found in the right hemisphere electrodes, which is not the case for the TD children in Panel C. Moreover, the timing of the stimulus differences in Panels B and C are different for the two groups of children, with the children with PD demonstrating much earlier stimulus differences. These earlier differences could be due more to acoustic-level processing, rather than phonological-level processing. This could indicate that these children are attending more to the acoustic differences of the stimuli, rather than the more relevant phonological feature information of the phonemes. Thus, there is some evidence that children with PD recruit atypical neural networks during speech processing tasks.

As this is the first study to address neural indices of phonological underspecification in children, caution should be taken to avoid over-interpretation. Although it is possible that the results could be due to the acoustic differences between the stimuli, the findings are consistent with an underspecification model. Future studies could address how the effects of acoustic differences can be distinguished from the effects of phonological underspecification in typical and atypical phonological development. This could be accomplished by comparing ERP responses elicited by phonemes to pure tones, or other non-linguistic stimuli, in TD children and children with PD.

### Conclusion

FUL predicts that [coronal] phonemes have the default place of articulation because they contain less distinctive feature information in their phonological representations than phonemes with other places of articulation. However, these language universal underspecification claims had not been tested in developmental populations until now. Neither the TD children, nor the children with PD, demonstrated the FUL-predicted mismatch asymmetry patterns seen in adult data. In fact, the children with PD demonstrated the exact opposite mismatch response pattern from that of adults, while TD children demonstrated mismatch responses to both specified and unspecified phonemes. Moreover, while both groups of children demonstrated similar responses to the underspecified /dɑ/, their neural responses to the more specified /bɑ/ varied. Thus, the children with PD did not appear to have the same level of phonetic information, or specification, in their phonological representations as TD children. These findings were interpreted within a proposed developmental model of phonological underspecification, wherein children with PD are functioning at a developmentally less mature stage of phonological acquisition than their same-aged TD peers. Thus, phonological specification, and underspecification, are phenomena that that likely develop over time with experience and exposure to language.

## Data Availability Statement

The raw data supporting the conclusions of this article will be made available by the authors, without undue reservation.

## Ethics Statement

The studies involving human participants were reviewed and approved by Idaho State University Human Subjects Committee and the University of North Dakota Institutional Review Board. Written informed consent to participate in this study was provided by the participants' legal guardian/next of kin.

## Author Contributions

AC created the stimuli, tested participants, prepared and analyzed the data, and helped write the manuscript. DO and YW helped analyze the data and write the manuscript. All authors contributed to the article and approved the submitted version.

## Conflict of Interest

The authors declare that the research was conducted in the absence of any commercial or financial relationships that could be construed as a potential conflict of interest.

## Footnotes

^1^These electrodes encompass four anterior-posterior levels (Frontal, Frontal-Central, Central, Central-Parietal) and seven left-right laterality levels (Far Left-5, Mid Left-3, Close Left-1, Midline-z, Close Right-2, Mid Right-4, Far Right-6).
