# Supplementary material for: Evidence for [Coronal] Underspecification in Typical and Atypical Phonological Development
Source: Front Hum Neurosci. 2020 Dec 22;14:580697. doi: 10.3389/fnhum.2020.580697 (PMC7782969; doi:10.3389/fnhum.2020.580697)

Supplementary Material

Supplementary Figure 1. Single-trial LIMO analyses data for participants #1-4. For each participant, statistically significant (uncorrected threshold) model R^2 at all electrodes in the timeline from 0 to 400 ms post-syllable onset with all electrodes stacked on the y-axis. Each figure is separated into four parts representing the four standard and deviant syllable analyses: A) /bɑ/ deviants and standards, B) /dɑ/ deviants and standards, C) /bɑ/ standards and /dɑ/ standards, and D) /bɑ/ deviants and /dɑ/ deviants.

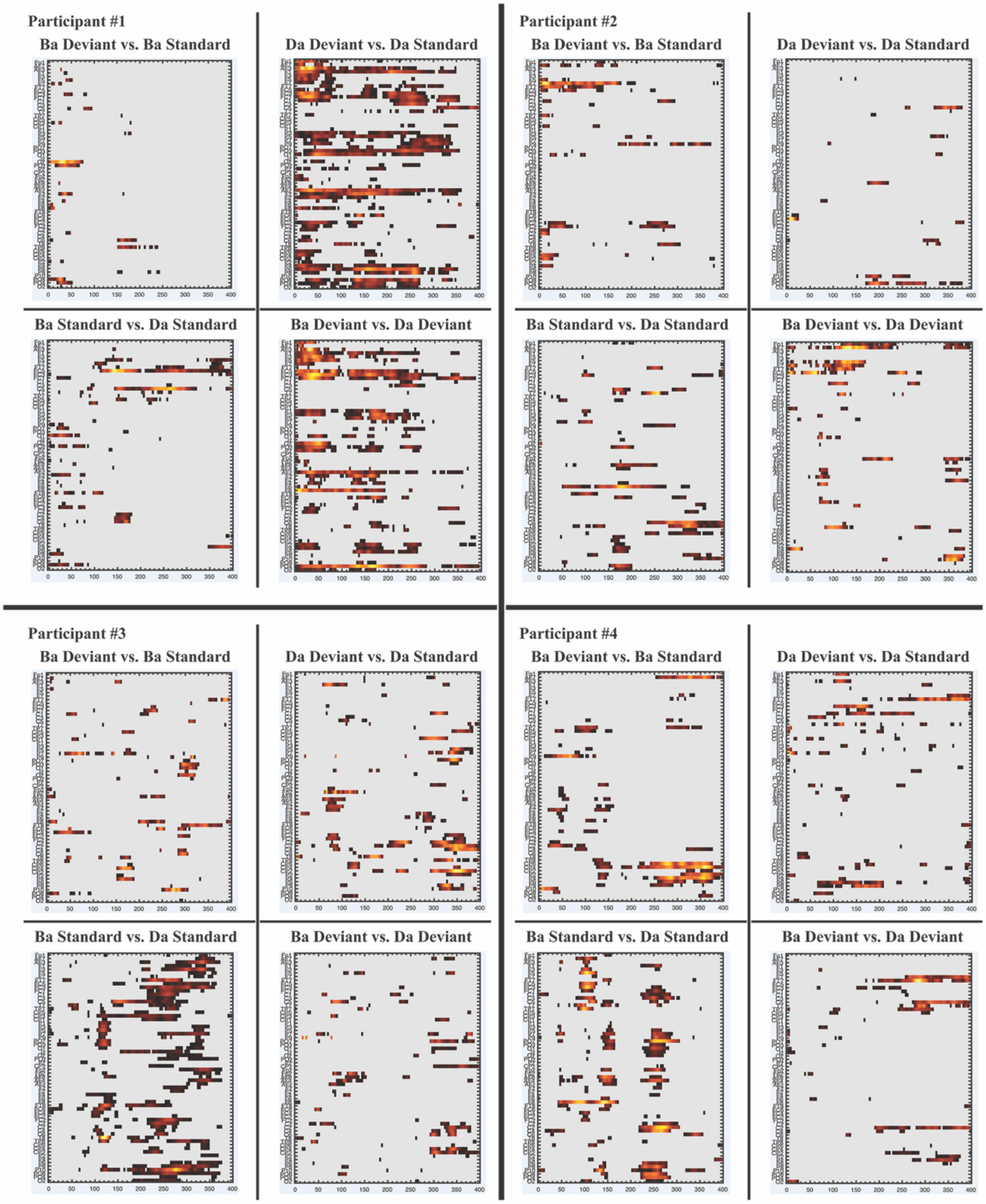

Supplementary Figure 2. Single-trial LIMO analyses data for participants #5-8. See caption for Supplementary Figure 1 for additional figure information.

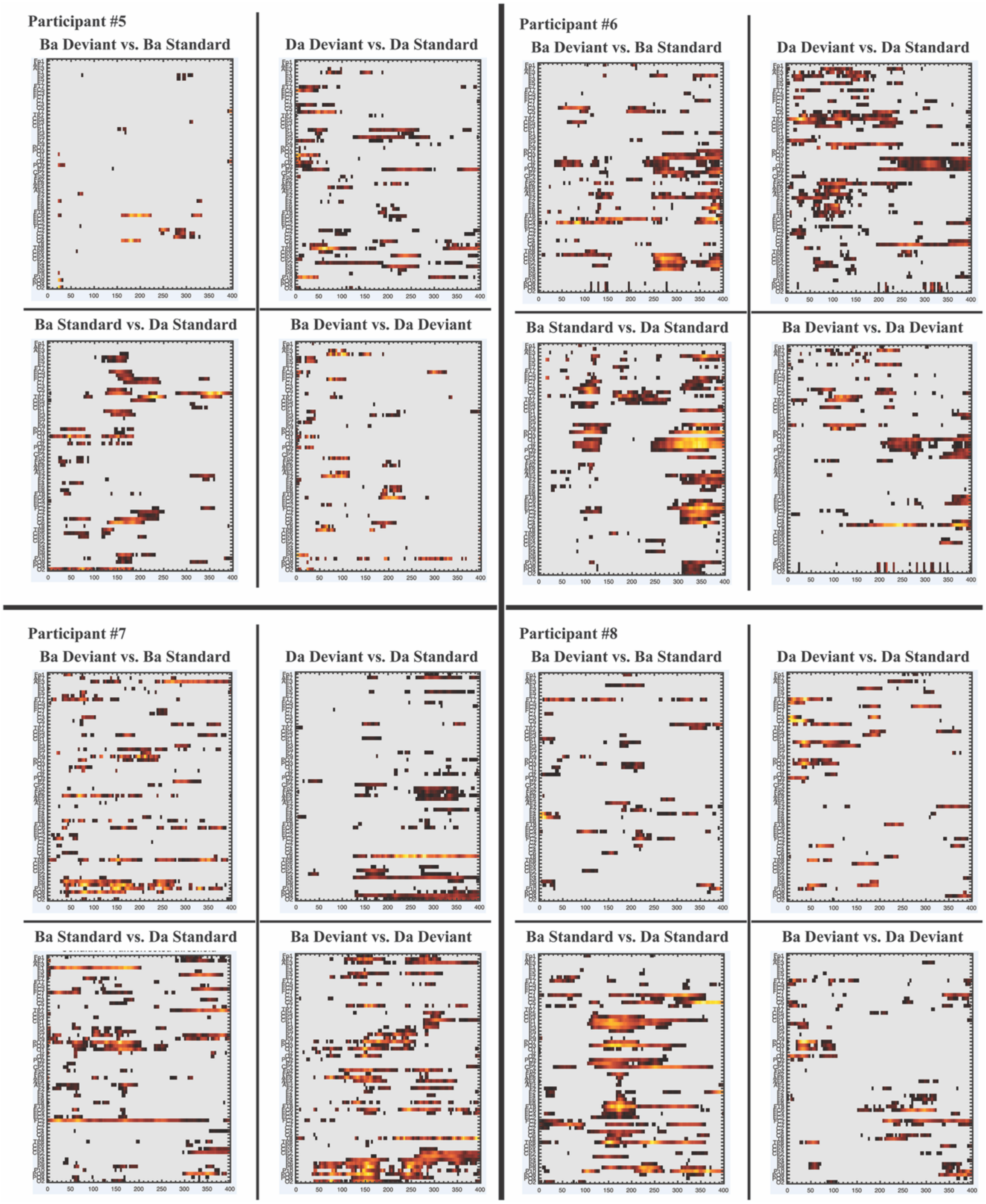

Supplementary Figure 3. Single-trial LIMO analyses data for participants #9-12. See caption for Supplementary Figure 1 for additional figure information.

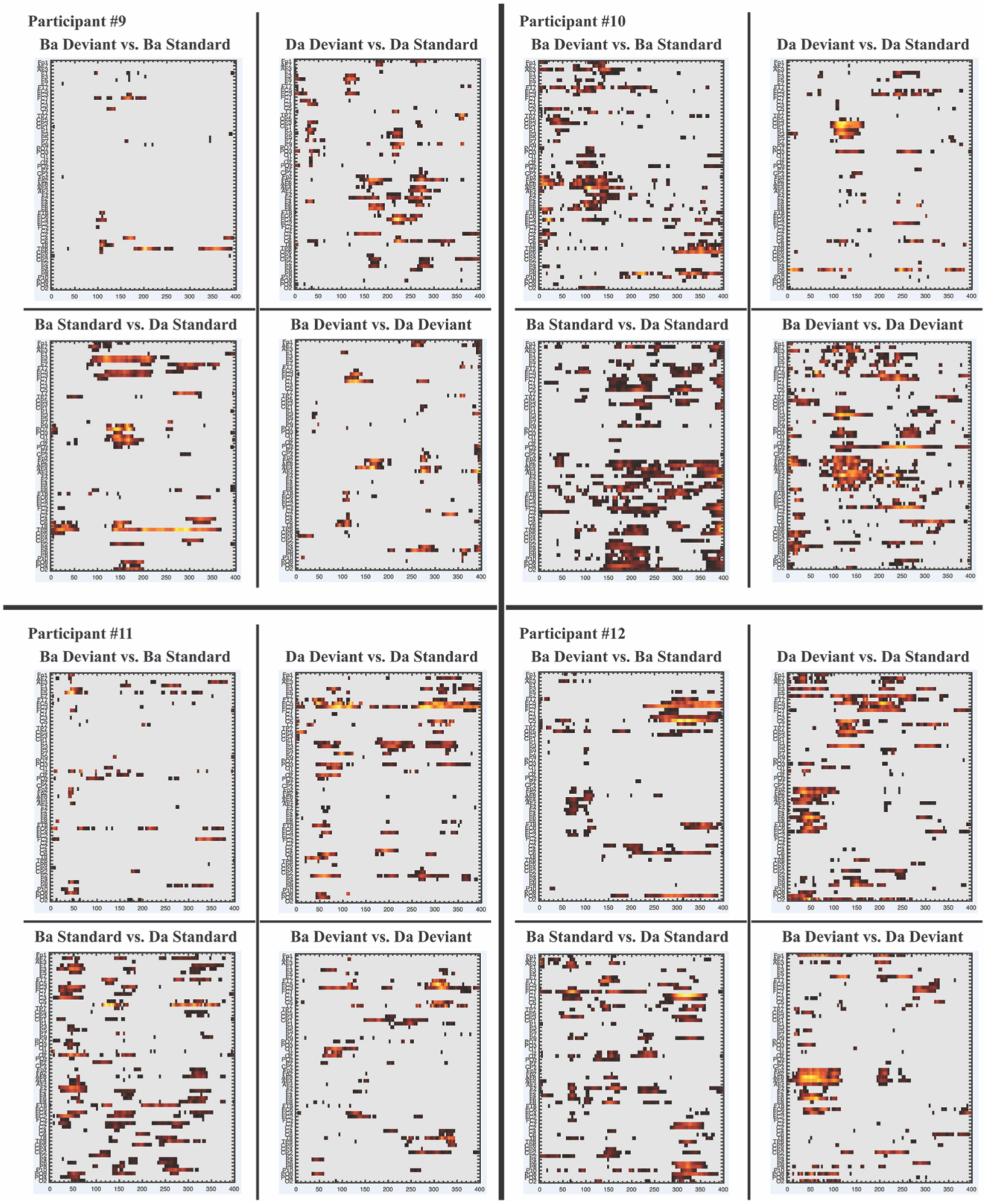

Supplementary Figure 4. Single-trial LIMO analyses data for participants #13-16. See caption for Supplementary Figure 1 for additional figure information.

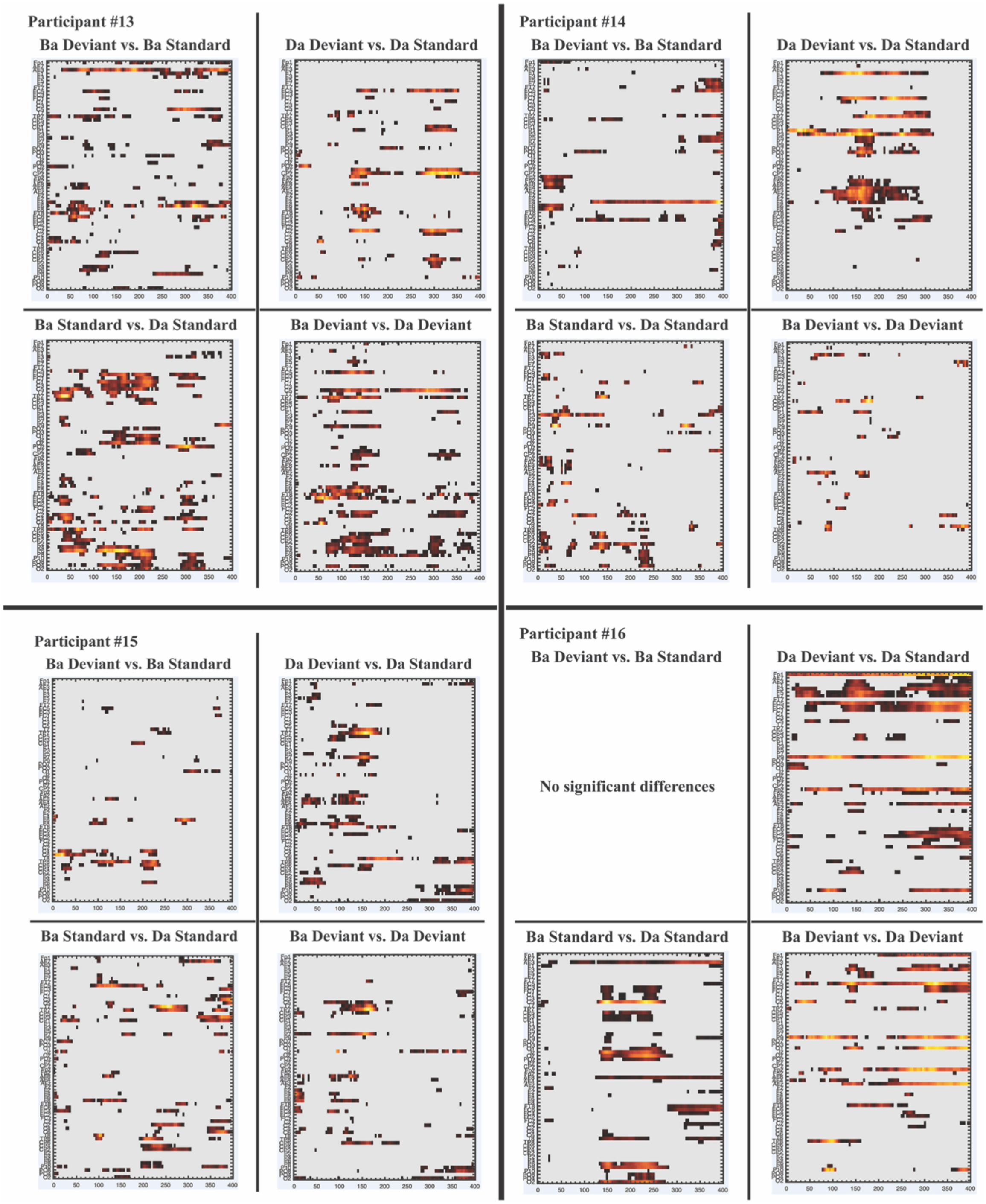

Supplementary Figure 5. Single-trial LIMO analyses data for participants #17-20. See caption for Supplementary Figure 1 for additional figure information.

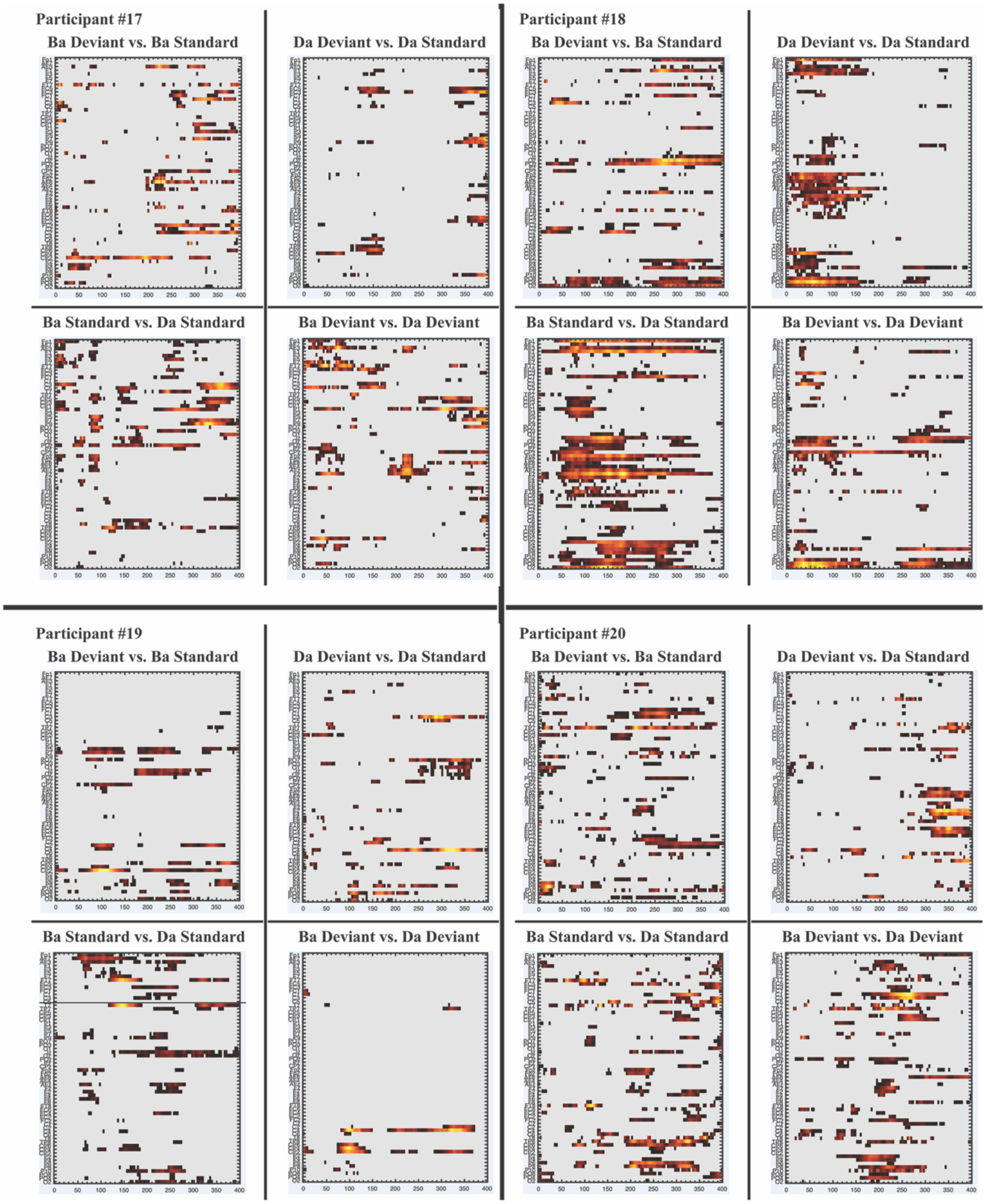

Supplementary Figure 6. Single-trial LIMO analyses data for participants #21-24. See caption for Supplementary Figure 1 for additional figure information.s

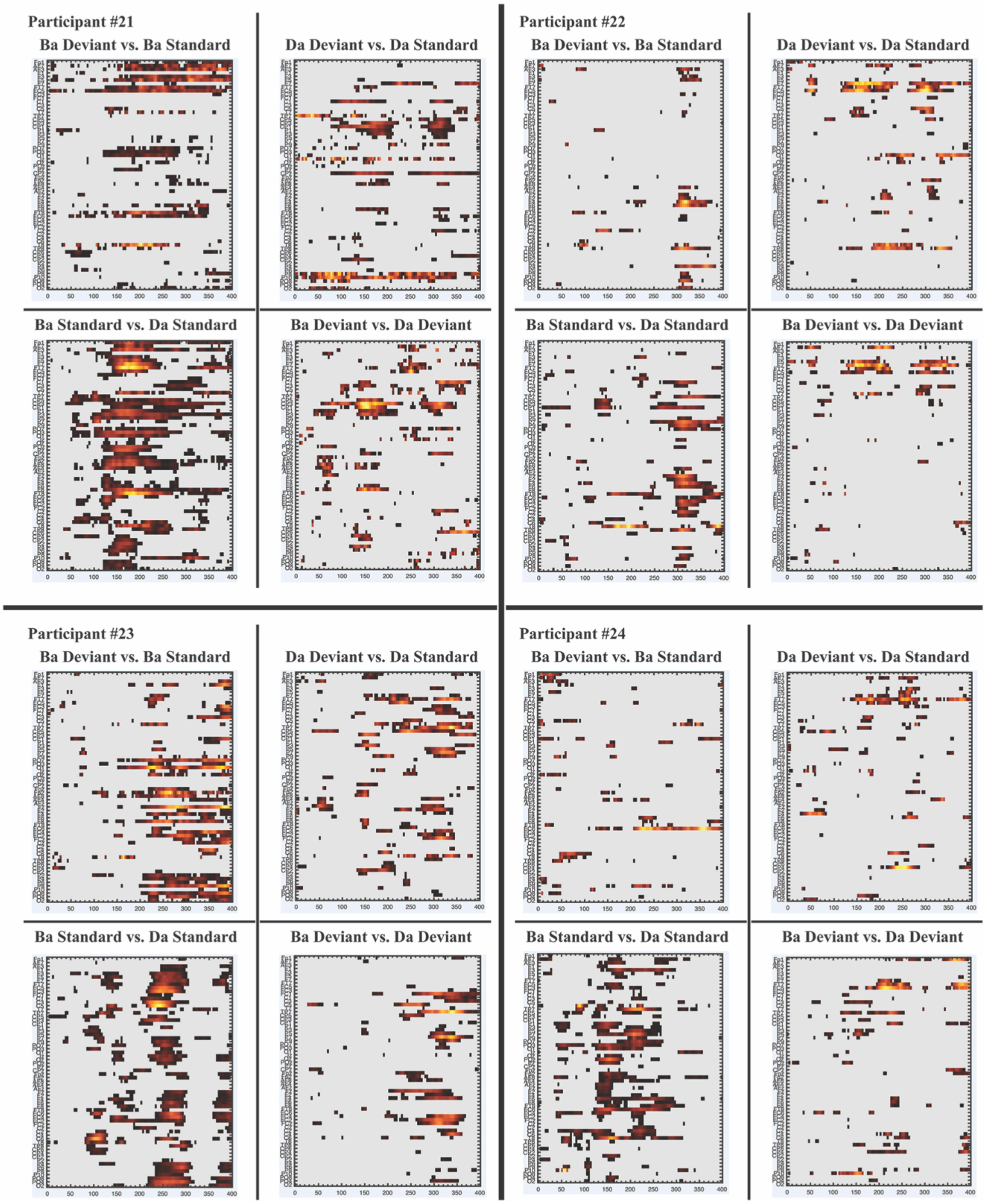

Supplement: Supplementary file 1 [file Data_Sheet_1.PDF]
